# Supplementary material for: Novel Purine Chemotypes with Activity against Plasmodium falciparum and Trypanosoma cruzi
Source: Pharmaceuticals (Basel). 2021 Jul 1;14(7):638. doi: 10.3390/ph14070638 (PMC8308784; doi:10.3390/ph14070638)
Supplement: Supplementary file 1 [file pharmaceuticals-14-00638-s001.zip › Supporting_information_revised.pdf]

Supporting information. ^{1}H-NMR, ^{13}C-NMR and HRMS data of compounds 1B, 2A, 3F, 4, 4M, 13H, 13ME, 21, 22, 23, 27, 28, 29, MJ-1, MJ-5, MJ-7, MJ-8, MJ-11, MJ-16, MJ-17, MJ-24, 6g, 6D. 1H NMR, 13C NMR, DEPT 135° and DEPTQ 135° spectra were recorded on a VarianInova Unity (300 MHz), BRUKER Nanobay Avance III HD (400 MHz) or BRUKER Avance NEO (400 or 500 MHz) spec-trometers and were internally referenced using residual protic solvent ($CDCl_3$: 1H NMR = 7.26, 13C NMR = 77.16). Chemical shifts are reported in parts per million (ppm, δ) downfield from tetramethylsilane (TMS). Coupling constants (J) are reported in Hz. Spin multiplicities are described as s (singlet), bs (broad singlet), d (doublet), t (triplet), q (quartet) and m (multiplet) or combinations of these terms. High-resolution mass spectra (HRMS) were recorded on a Waters LCT Premier XE Spectrometer.

N,9-Di-*tert*-butyl-8-phenyl-9*H*-purin-6-amine (**1B**). ^{1}H NMR (400 MHz, $CDCl_3$) δ 8.39 (s, 1H), 7.52 – 7.40 (m, 6H), 5.78 (bs, 1H), 1.63 (s, 9H), 1.55 (s, 9H). ^{13}C NMR (101 MHz, $CDCl_3$) δ 151.30, 129.95, 129.59, 128.22, 60.46, 52.28, 49.82, 31.03, 29.31. HRMS (ES + ve), $C_{19}H_{26}N_5$ $(M + H)^+$: Calculated 324.2188. Obtained 324.2164.

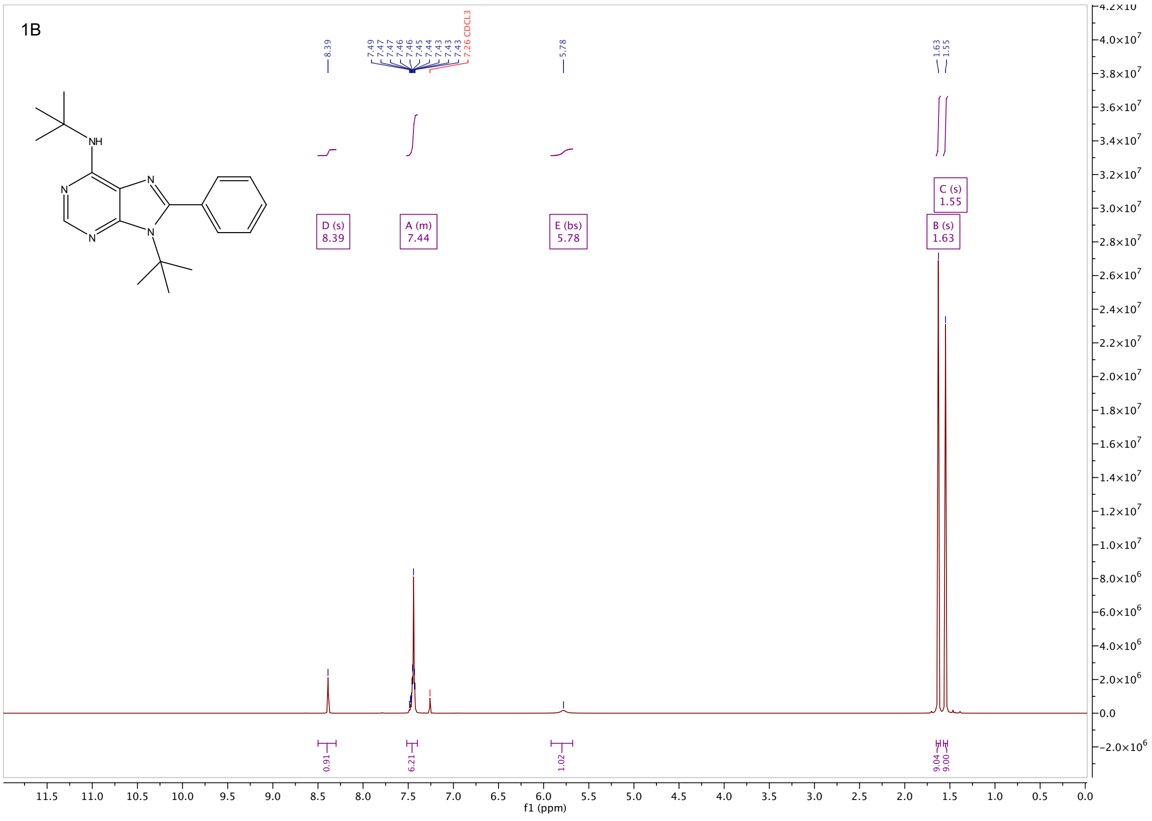

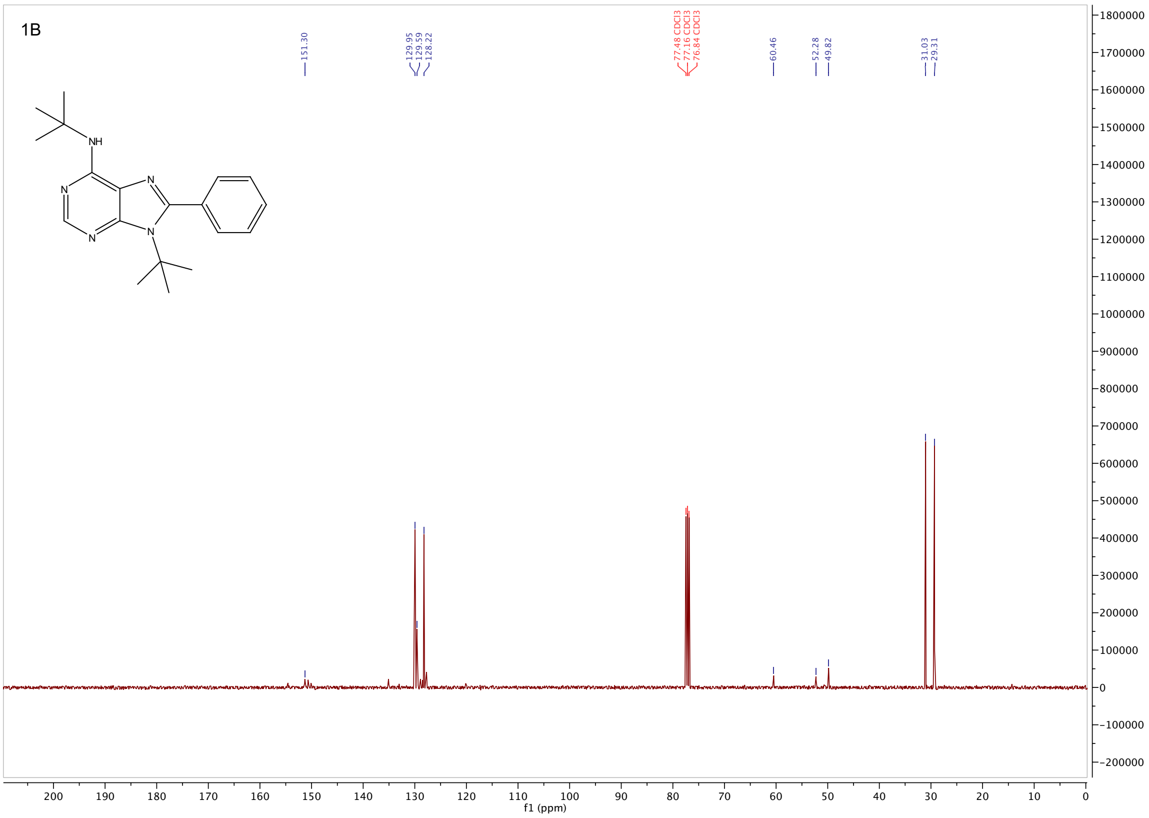

Elemental Composition Report **Page 1**

Single Mass Analysis
Tolerance = 10.0 PPM / DBE: min = -1.5, max = 50.0
Element prediction: Off
Number of isotope peaks used for i-FIT = 3

Monoisotopic Mass, Even Electron Ions
65 formula(e) evaluated with 2 results within limits (up to 50 best isotopic matches for each mass)
Elements Used:
C: 0-19 H: 0-1000 N: 0-5 Na: 0-1 I: 0-2

MJ-di1B 41 (0.900) AM (Cen,6, 100.00, Ar,5000.0,0.00,1.00)
1: TOF MS ES+

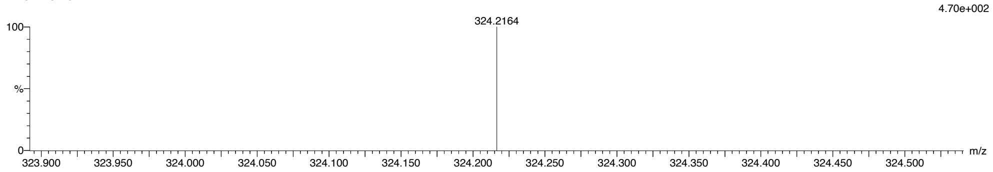

Minimum:				-1.5			
Maximum:		5.0	10.0	50.0			
Mass	Calc. Mass	mDa	PPM	DBE	i-FIT	i-FIT (Norm)	Formula
324.2164	324.2188	-2.4	-7.4	9.5	23.0	1.4	C19 H26 N5
	324.2164	0.0	0.0	6.5	21.8	0.3	C17 H27 N5 Na

N,9-Di-*tert*-butyl-8-methyl-9*H*-purin-6-amine (**2A**). ^{1}H NMR (400 MHz, $CDCl_3$) δ 8.30 (s, 1H), 5.69 (bs, 1H), 2.73 (s, 3H), 1.85 (s, 9H), 1.55 (s, 9H). ^{13}C NMR (101 MHz, $CDCl_3$) δ 153.90, 151.15, 150.85, 147.99, 123.57, 60.04, 52.20, 30.56, 29.35, 19.99. HRMS (ES + ve), $C_{14}H_{24}N_5$ $(M + H)^+$: Calculated 262.2032. Obtained 262.2029.

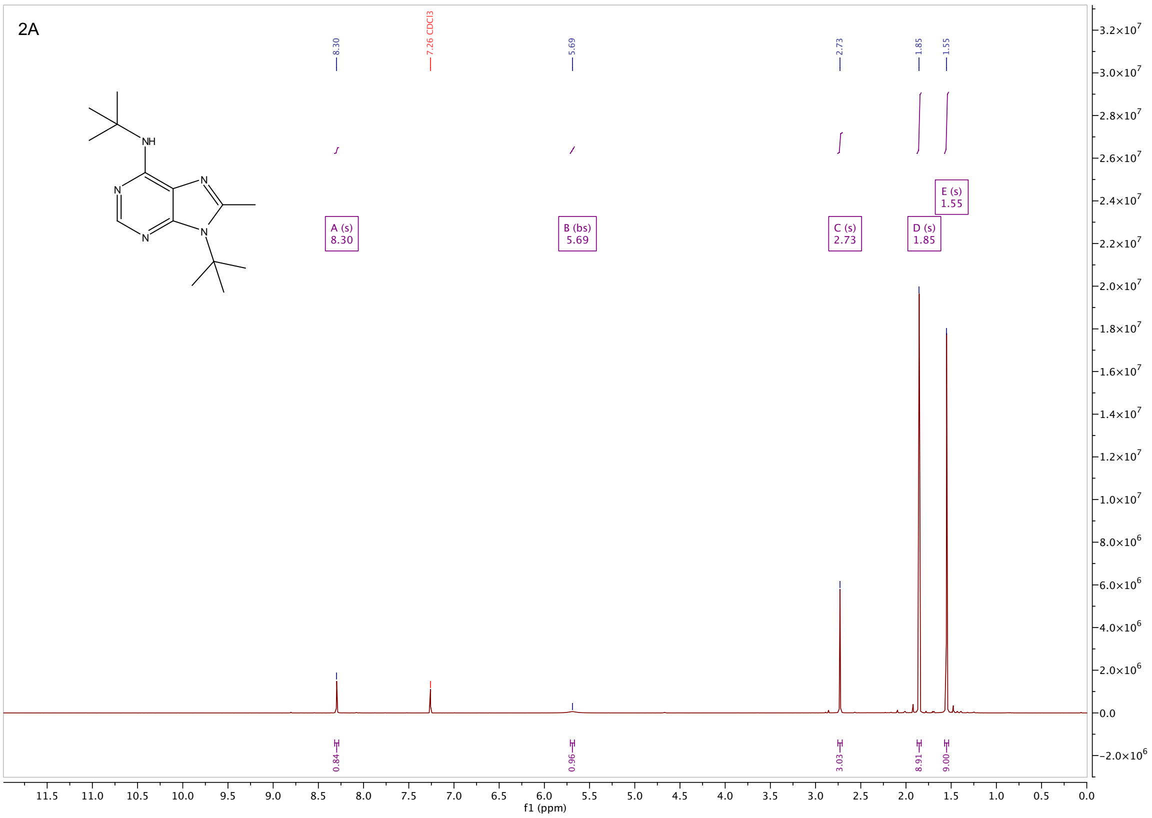

2A

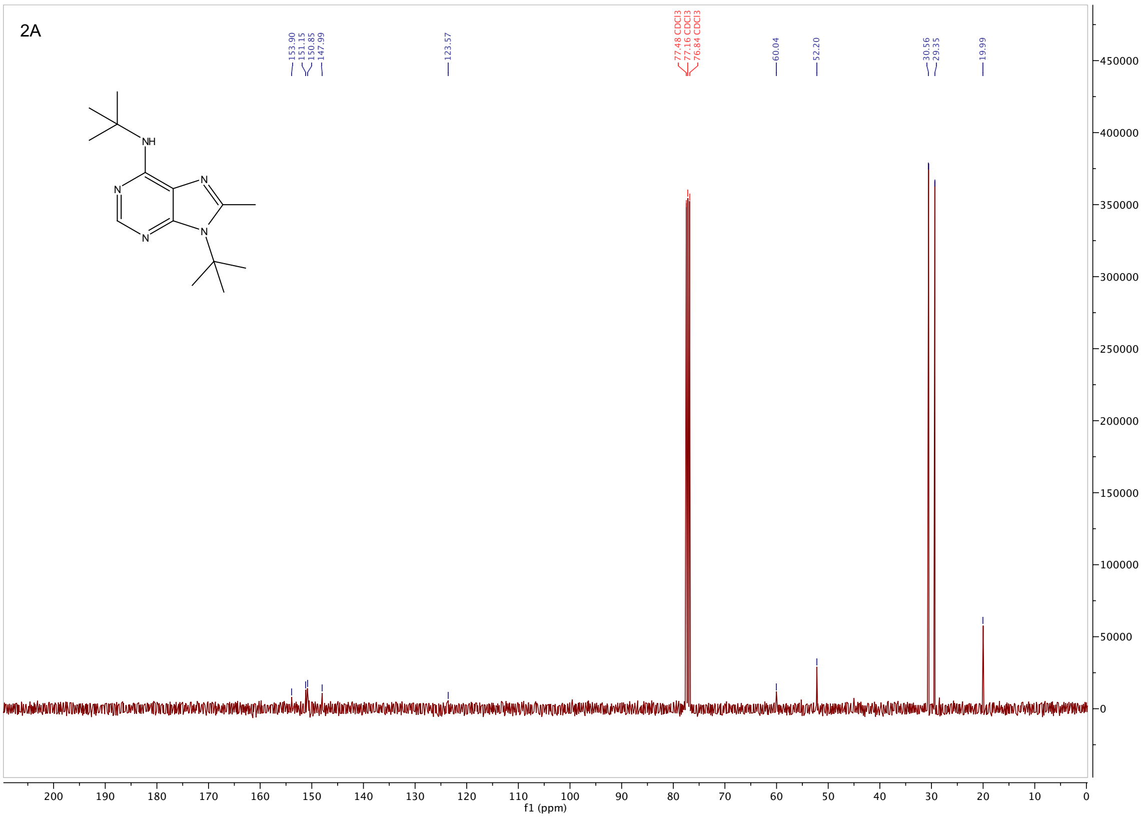

Elemental Composition Report **Page 1**

Single Mass Analysis
Tolerance = 10.0 PPM / DBE: min = -1.5, max = 50.0
Element prediction: Off
Number of isotope peaks used for i-FIT = 3

Monoisotopic Mass, Even Electron Ions
25 formula(e) evaluated with 2 results within limits (up to 50 best isotopic matches for each mass)
Elements Used:
C: 0-14 H: 0-1000 N: 0-6 Na: 0-1
MJ-di2-2 162 (3.544) AM (Top,1, Ar,5000.0,0.00,1.00)
1: TOF MS ES+

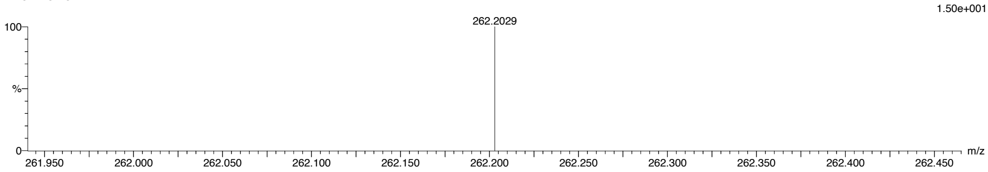

Minimum:				-1.5			
Maximum:		5.0	10.0	50.0			
Mass	Calc. Mass	mDa	PPM	DBE	i-FIT	i-FIT (Norm)	Formula
262.2029	262.2032	-0.3	-1.1	5.5	12.3	0.7	C14 H24 N5
	262.2008	2.1	8.0	2.5	12.2	0.7	C12 H25 N5 Na

N,9-Di-*tert*-butyl-9*H*-purin-6-amine (**3F**). ^{1}H NMR (400 MHz, $CDCl_3$) δ 8.34 (s, 1H), 7.76 (s, 1H), 5.71 (bs, 1H), 1.76 (s, 9H), 1.55 (s, 9H). 13C NMR (101 MHz, $CDCl_3$) δ 154.89, 151.62, 149.11, 136.88, 121.68, 57.24, 52.24, 29.30, 29.20. HRMS (ES + ve), C13H22N5 (M + H)+: Calculated 248.1875. Obtained 248.1862.

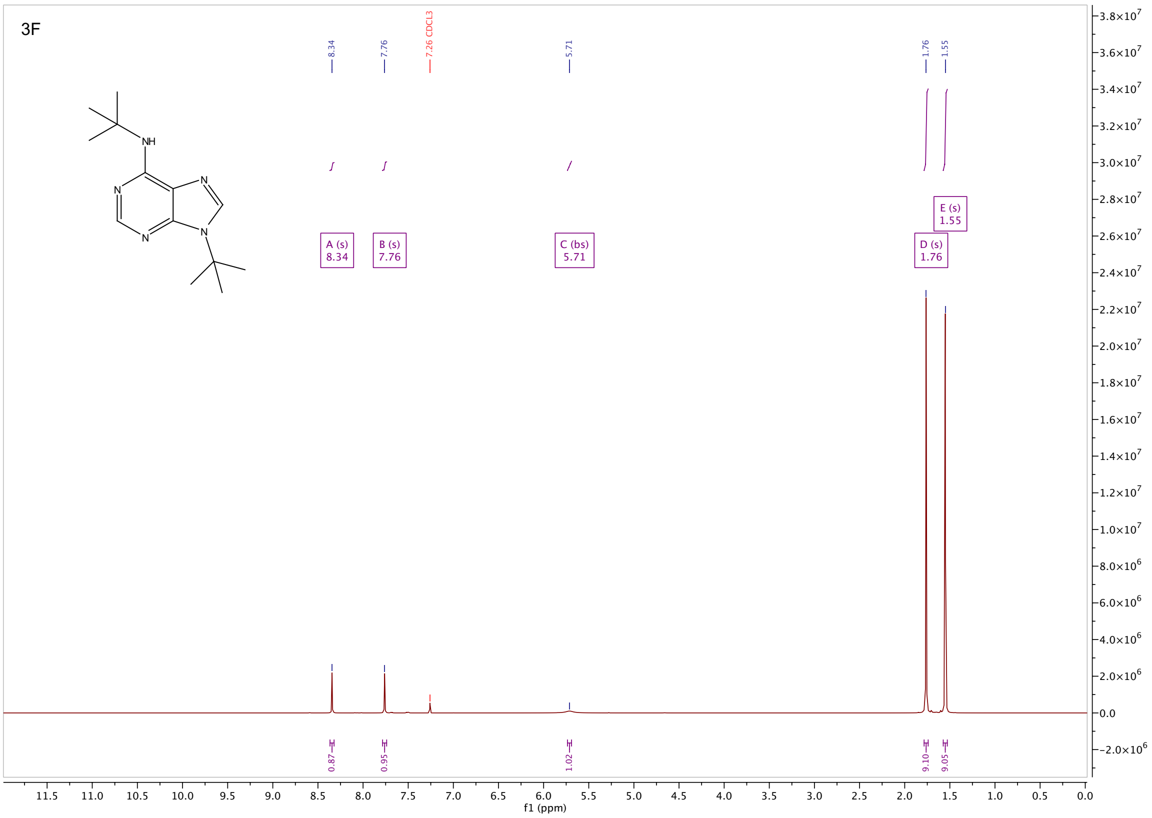

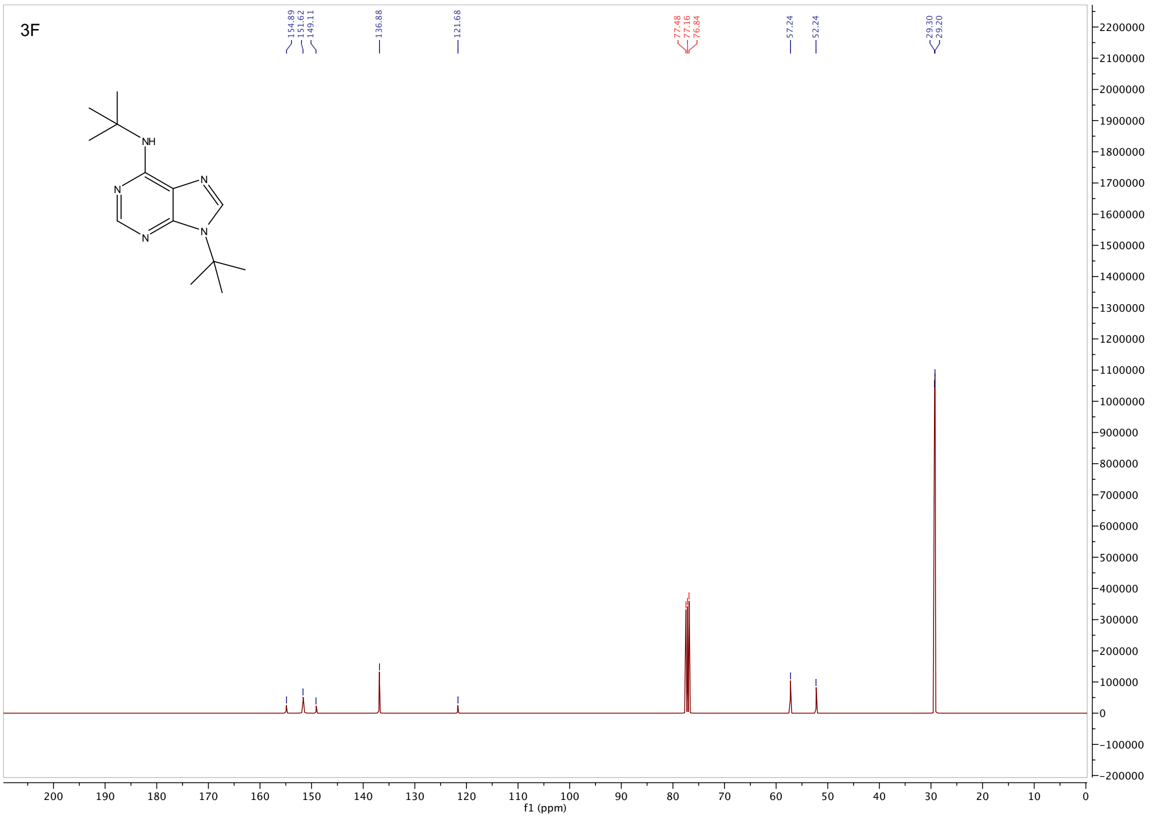

Elemental Composition Report **Page 1**

Single Mass Analysis
Tolerance = 10.0 PPM / DBE: min = -1.5, max = 50.0
Element prediction: Off
Number of isotope peaks used for i-FIT = 3

Monoisotopic Mass, Even Electron Ions
158 formula(e) evaluated with 2 results within limits (up to 50 best isotopic matches for each mass)
Elements Used:
C: 0-22 H: 0-1000 N: 0-5 O: 0-3 Na: 0-1
MJ-di3 45 (1.005) AM (Top,6, Ar,5000.0,0.00,1.00)
1: TOF MS ES+

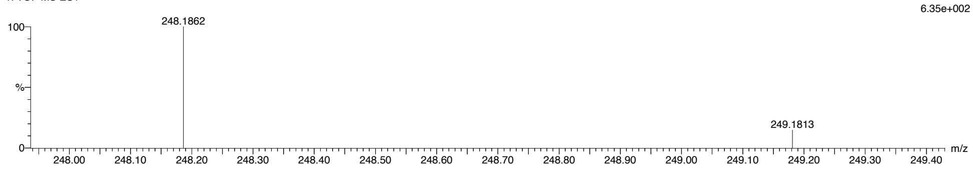

Mass	Calc. Mass	mDa	PPM	DBE	i-FIT	i-FIT (Norm)	Formula
Minimum:				-1.5			
Maximum:		5.0	10.0	50.0			
248.1862	248.1875	-1.3	-5.2	5.5	17.1	1.8	C13 H22 N5
	248.1851	1.1	4.4	2.5	15.4	0.2	C11 H23 N5 Na

6-(Benzyloxy)-*N*,*N*-dimethyl-8-phenyl-9*H*-purin-9-amine (**4**). ^{1}H NMR (500 MHz, $CDCl_3$) δ 8.50 (s, 1H), 8.25 – 8.18 (m, 2H), 7.59 – 7.53 (m, 2H), 7.52 – 7.43 (m, 3H), 7.39 – 7.34 (m, 2H), 7.34 – 7.28 (m, 1H), 5.71 (s, 2H), 3.23 (s, 6H). ^{13}C NMR (126 MHz, $CDCl_3$) δ 160.45, 153.26, 151.06, 150.77, 136.51, 130.44, 129.56, 129.19, 128.68, 128.55, 128.37, 128.25, 120.10, 68.41, 45.56. HRMS (ES + ve), $C_{20}H_{20}N_5O$ (M + H)$^{+}$: Calculated 346.1668. Obtained 346.1636.

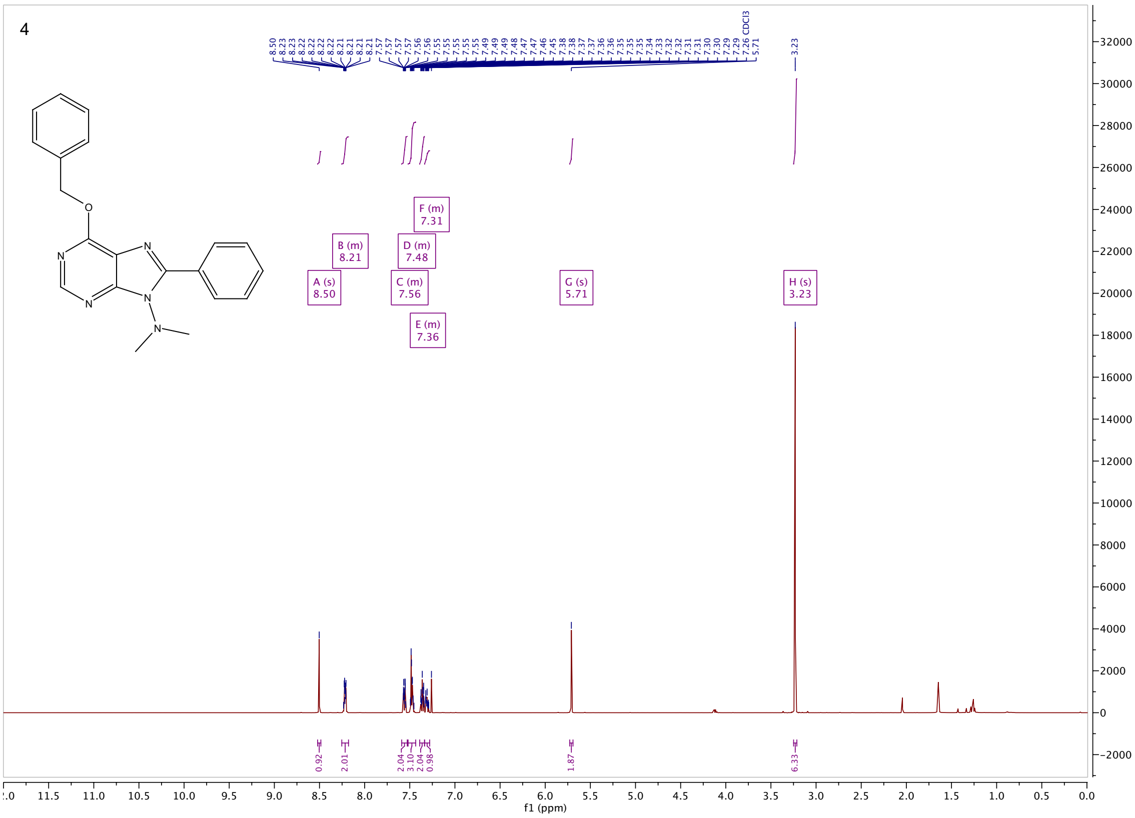

4

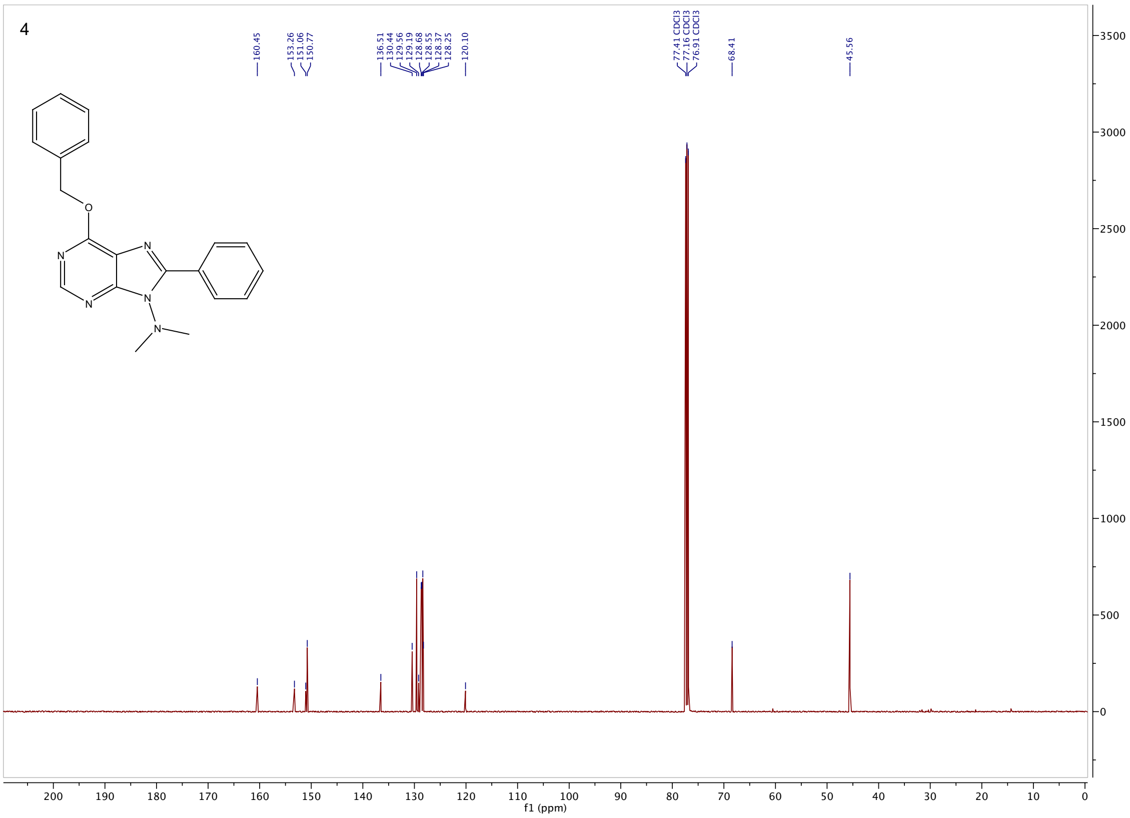

Elemental Composition Report **Page 1**

Single Mass Analysis
Tolerance = 10.0 PPM / DBE: min = -1.5, max = 50.0
Element prediction: Off
Number of isotope peaks used for i-FIT = 3

Monoisotopic Mass, Even Electron Ions
192 formula(e) evaluated with 2 results within limits (up to 50 best isotopic matches for each mass)
Elements Used:
C: 0-38 H: 0-1000 N: 0-8 O: 0-4
ALM-4-1 12 (0.229) AM (Cen,6, 100.00, Ar,5000.0,0.00,1.00)
1: TOF MS ES+

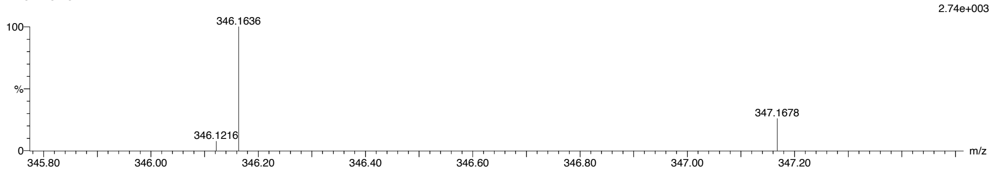

Minimum:				-1.5			
Maximum:		5.0	10.0	50.0			
Mass	Calc. Mass	mDa	PPM	DBE	i-FIT	i-FIT (Norm)	Formula
346.1636	346.1668	-3.2	-9.2	13.5	23.9	1.5	C20 H20 N5 O
	346.1628	0.8	2.3	9.5	22.7	0.3	C15 H20 N7 O3

6-(Benzyloxy)-*N*,*N*,8-trimethyl-9*H*-purin-9-amine (**4M**). ^{1}H NMR (400 MHz, $CDCl_3$) δ 7.89 (s, 1H), 7.37 – 7.25 (m, 5H), 5.24 (s, 2H), 3.06 (s, 6H), 2.48 (s, 3H). ^{13}C NMR (101 MHz, $CDCl_3$) δ 156.42, 149.69, 147.64, 145.13, 136.36, 129.07, 128.32, 128.30, 122.10, 49.00, 46.05, 13.30. HRMS (ES + ve), $C_{15}H_{18}N_5O$ $(M + H)^+$: Calculated 284.1511. Obtained 284.1504.

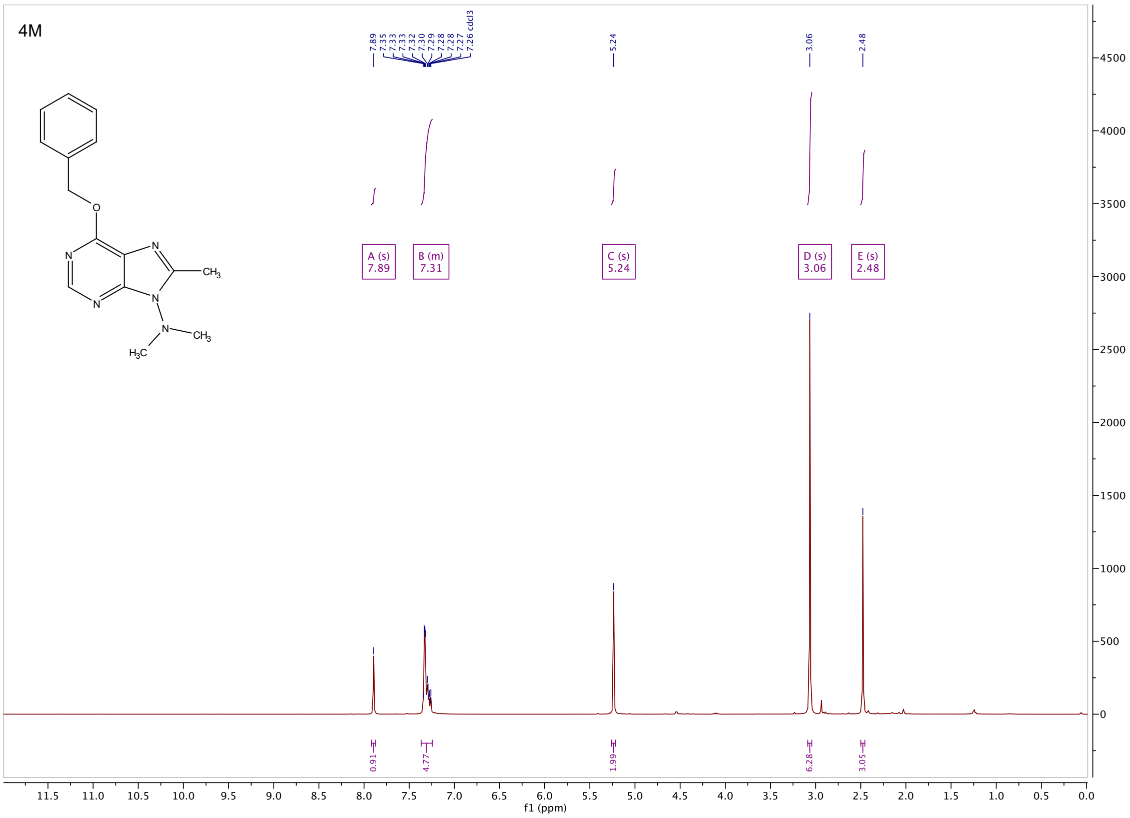

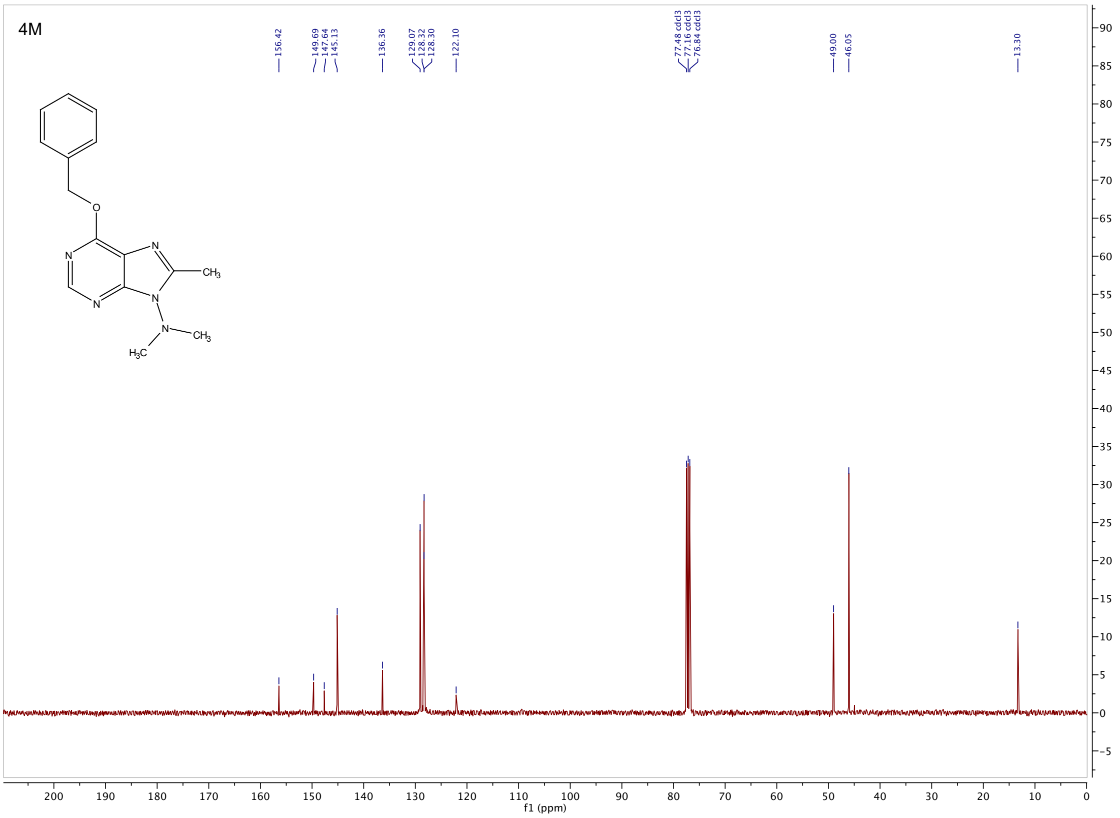

Elemental Composition Report **Page 1**

Single Mass Analysis
Tolerance = 10.0 PPM / DBE: min = -1.5, max = 50.0
Element prediction: Off
Number of isotope peaks used for i-FIT = 3

Monoisotopic Mass, Even Electron Ions
227 formula(e) evaluated with 2 results within limits (up to 50 closest results for each mass)
Elements Used:
C: 0-19 H: 0-1000 N: 0-6 O: 0-4 Na: 0-1
ALM8P2 12 (0.263) AM (Top,1, Ht,5000.0,0.00,1.00)
1: TOF MS ES+

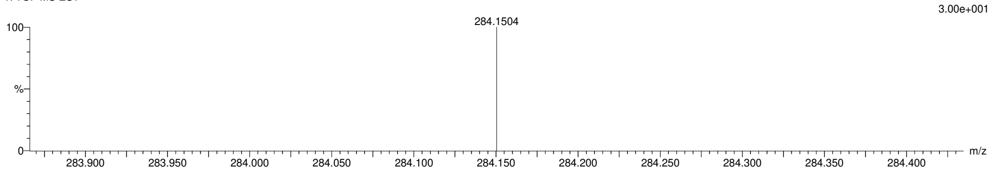

Minimum:				-1.5			
Maximum:		5.0	10.0	50.0			
Mass	Calc. Mass	mDa	PPM	DBE	i-FIT	i-FIT (Norm)	Formula
284.1504	284.1511	-0.7	-2.5	9.5	14.5	0.9	C15 H18 N5 O
	284.1487	1.7	6.0	6.5	14.2	0.6	C13 H19 N5 O Na

6-(Benzyloxy)-9-(piperidin-1-yl)-9*H*-purine (**13H**). ^{1}H NMR (400 MHz, $CDCl_3$) δ 7.98 (s, 1H), 7.87 (s, 1H), 7.39 – 7.24 (m, 5H), 5.24 (s, 2H), 3.39 – 3.32 (m, 4H), 1.83 – 1.73 (m, 4H), 1.60 – 1.50 (m, 2H). ^{13}C NMR (101 MHz, $CDCl_3$) δ 156.75, 146.81, 146.31, 139.35, 136.15, 129.12, 128.42, 128.32, 123.46, 56.50, 49.22, 26.16, 23.18. HRMS (ES + ve), $C_{17}H_{20}N_5O$ $(M + H)^+$: Calculated 310.1668. Obtained 310.1680.

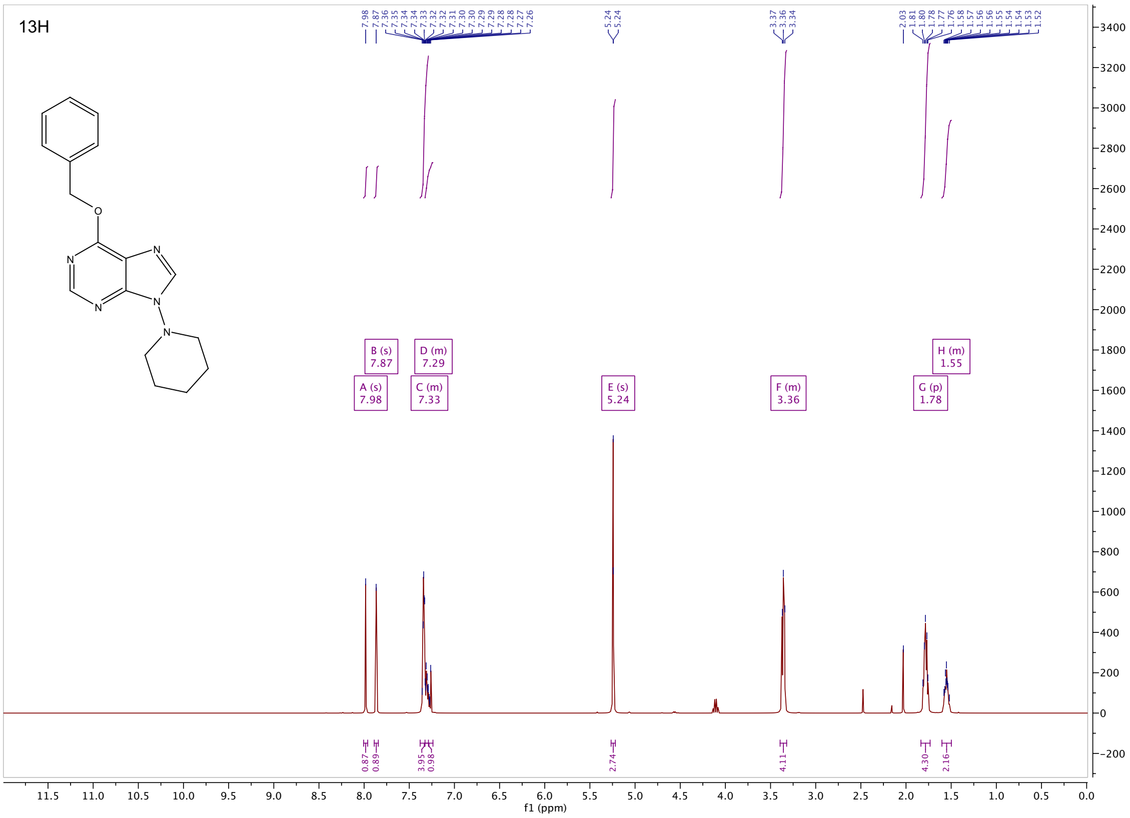

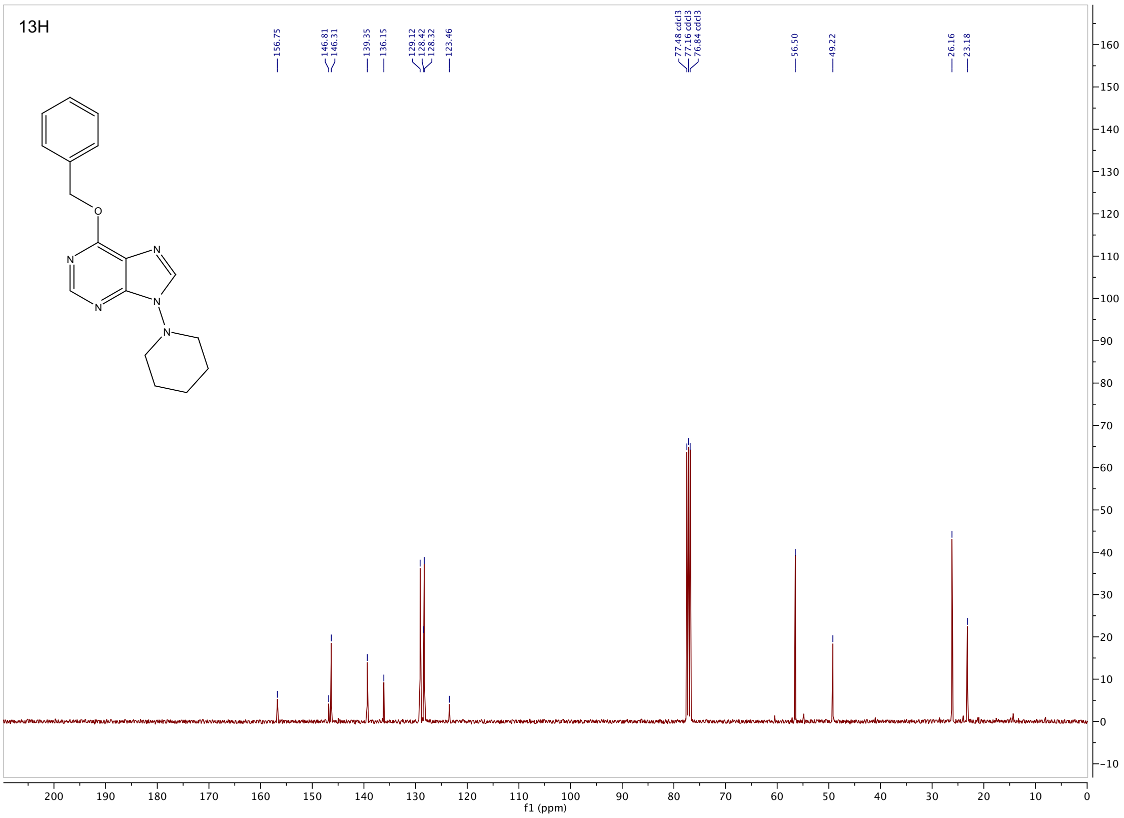

Elemental Composition Report **Page 1**

Single Mass Analysis

Tolerance = 10.0 PPM / DBE: min = -1.5, max = 50.0
Element prediction: Off
Number of isotope peaks used for i-FIT = 3

Monoisotopic Mass, Even Electron Ions
213 formula(e) evaluated with 1 results within limits (up to 50 closest results for each mass)
Elements Used:
C: 0-19 H: 0-1000 N: 0-6 O: 0-4 Na: 0-1
ALM10P3 29 (0.599) AM (Top,1, Ht,5000.0,0.00,1.00)
1: TOF MS ES+

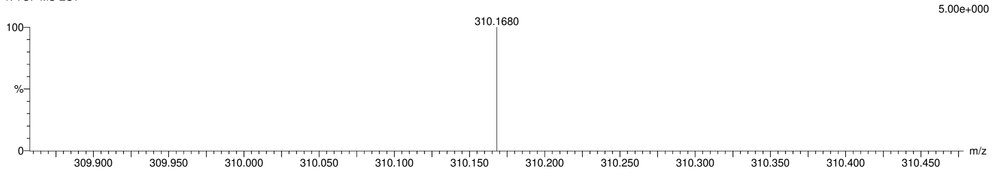

Minimum:				-1.5
Maximum:		5.0	10.0	50.0

Mass	Calc. Mass	mDa	PPM	DBE	i-FIT	i-FIT (Norm)	Formula
310.1680	310.1668	1.2	3.9	10.5	12.3	0.0	C17 H20 N5 O

6-(Benzyloxy)-8-methyl-9-(piperidin-1-yl)-9*H*-purine (**13ME**). ^{1}H NMR (400 MHz, $CDCl_3$) δ 7.88 (s, 1H), 7.37 – 7.24 (m, 5H), 5.23 (s, 2H), 3.75 – 3.67 (m, 2H), 3.10 – 3.04 (m, 2H), 2.48 (s, 3H), 1.86 – 1.62 (m, 6H). ^{13}C NMR (101 MHz, $CDCl_3$) δ 156.43, 149.88, 147.81, 144.95, 136.36, 129.07, 128.33, 128.31, 121.94, 54.86, 48.99, 26.44, 23.24, 13.28. HRMS (ES + ve), $C_{18}H_{22}N_5O$ $(M + H)^+$: Calculated 324.1824. Obtained 324.1844.

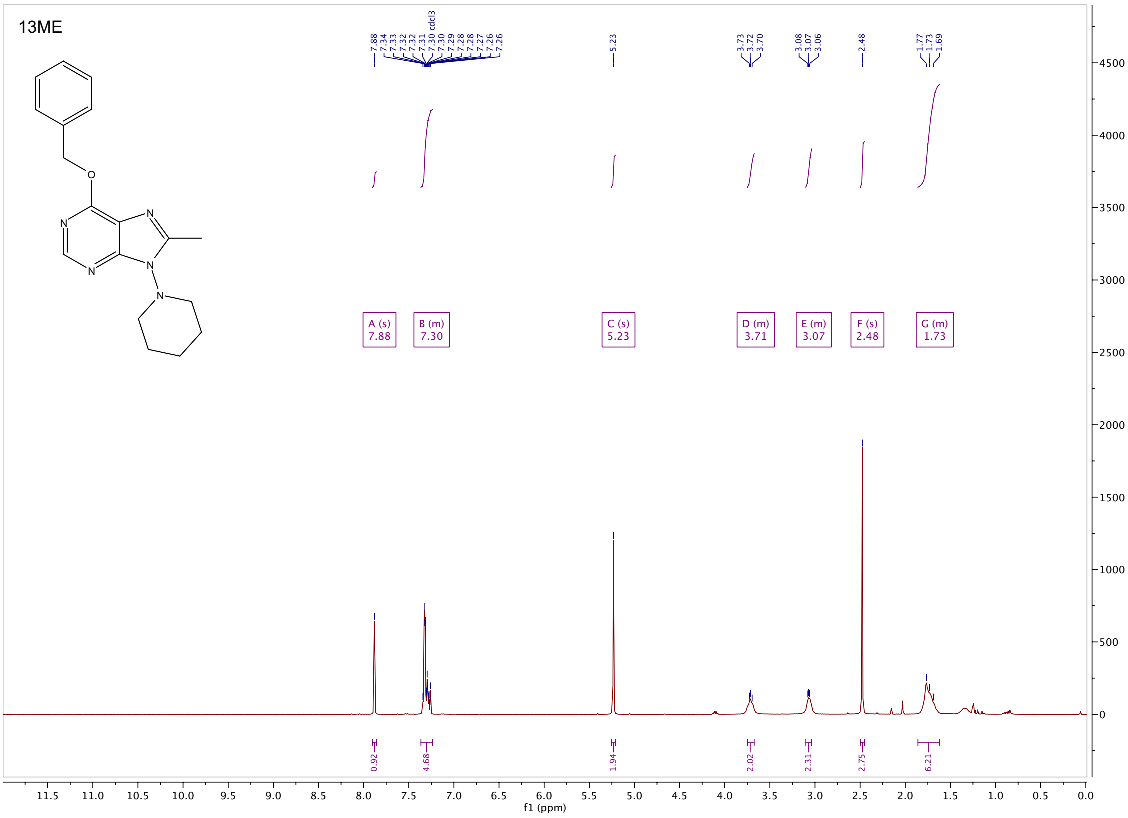

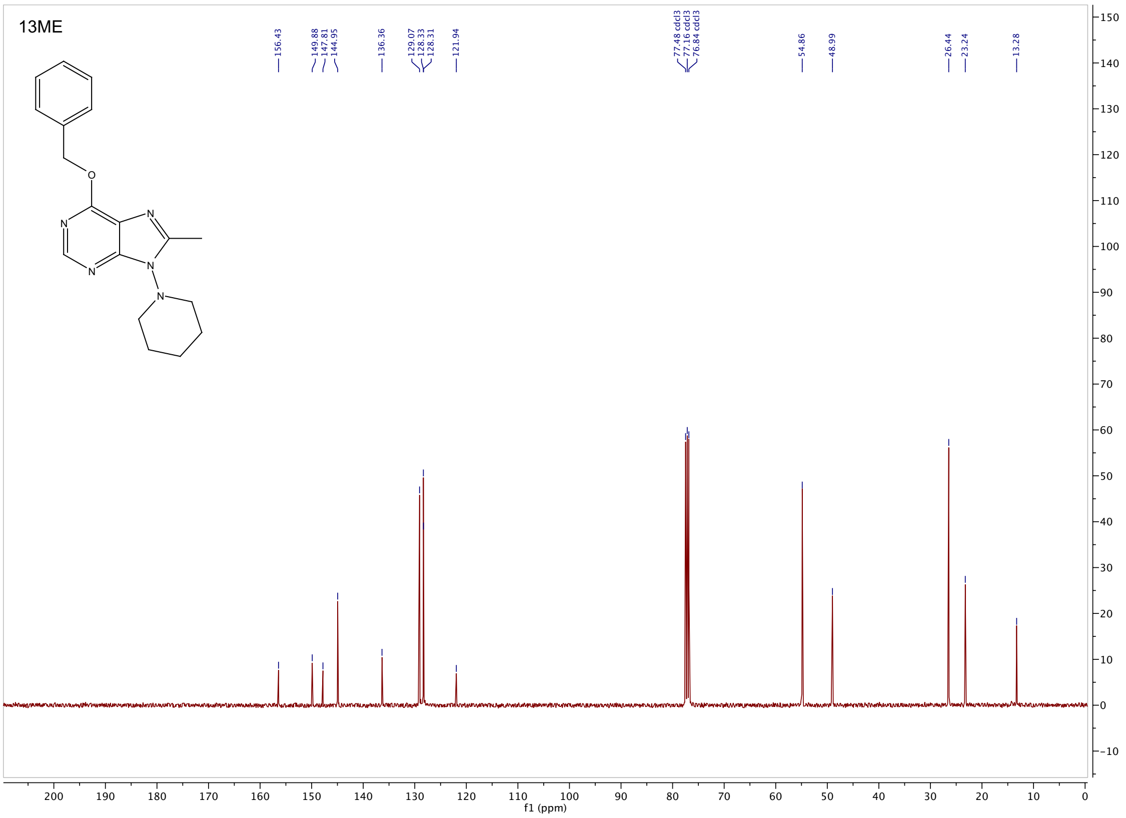

Elemental Composition Report **Page 1**

Single Mass Analysis
Tolerance = 10.0 PPM / DBE: min = -1.5, max = 50.0
Element prediction: Off
Number of isotope peaks used for i-FIT = 3

Monoisotopic Mass, Even Electron Ions
315 formula(e) evaluated with 2 results within limits (up to 50 closest results for each mass)
Elements Used:
C: 0-18 H: 0-1000 N: 0-5 O: 0-9 Na: 0-1
ALM10P2 136 (2.961) AM (Top,1, Ht,5000.0,0.00,1.00)
1: TOF MS ES+

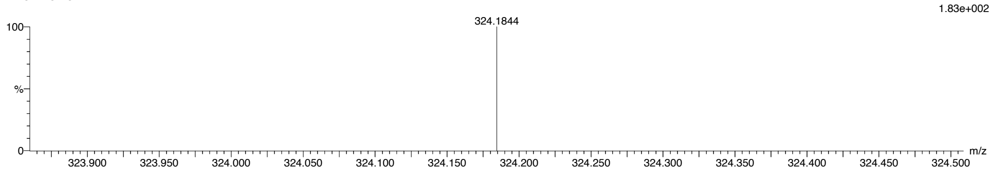

Minimum:				-1.5			
Maximum:		5.0	10.0	50.0			

Mass	Calc. Mass	mDa	PPM	DBE	i-FIT	i-FIT (Norm)	Formula
324.1844	324.1824	2.0	6.2	10.5	20.1	1.3	C18 H22 N5 O
	324.1859	-1.5	-4.6	-1.5	19.1	0.3	C9 H27 N5 O6 Na

4-(6-(Benzyloxy)-8-phenyl-9*H*-purin-9-yl)morpholine (**21**). ^{1}H NMR (500 MHz,) δ 8.50 (s, 1H), 8.25 – 8.17 (m, 2H), 7.59 – 7.53 (m, 2H), 7.49 (dd, *J* = 5.2, 2.0 Hz, 3H), 7.40 – 7.34 (m, 2H), 7.34 – 7.27 (m, 1H), 5.71 (s, 2H), 4.39 (s, 2H), 3.92 (t, *J* = 48.4 Hz, 4H), 3.06 (s, 2H). ^{13}C NMR (126 MHz, $CDCl_3$) δ 160.50, 153.29, 150.97, 150.91, 136.44, 130.53, 129.58, 128.98, 128.71, 128.55, 128.37, 128.27, 119.96, 68.46, 67.17, 53.46. HRMS (ES + ve), $C_{22}H_{22}N_5O_2$ $(M + H)^+$: Calculated 388.1774. Obtained 388.1761.

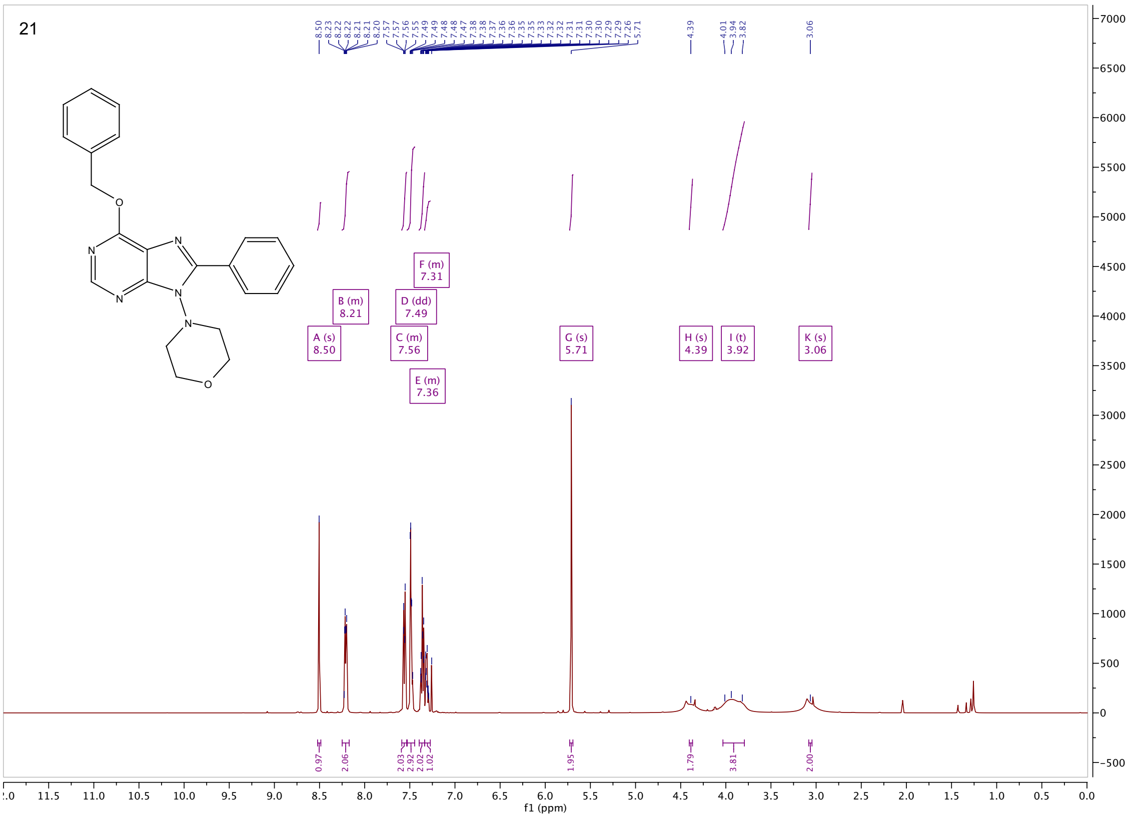

21

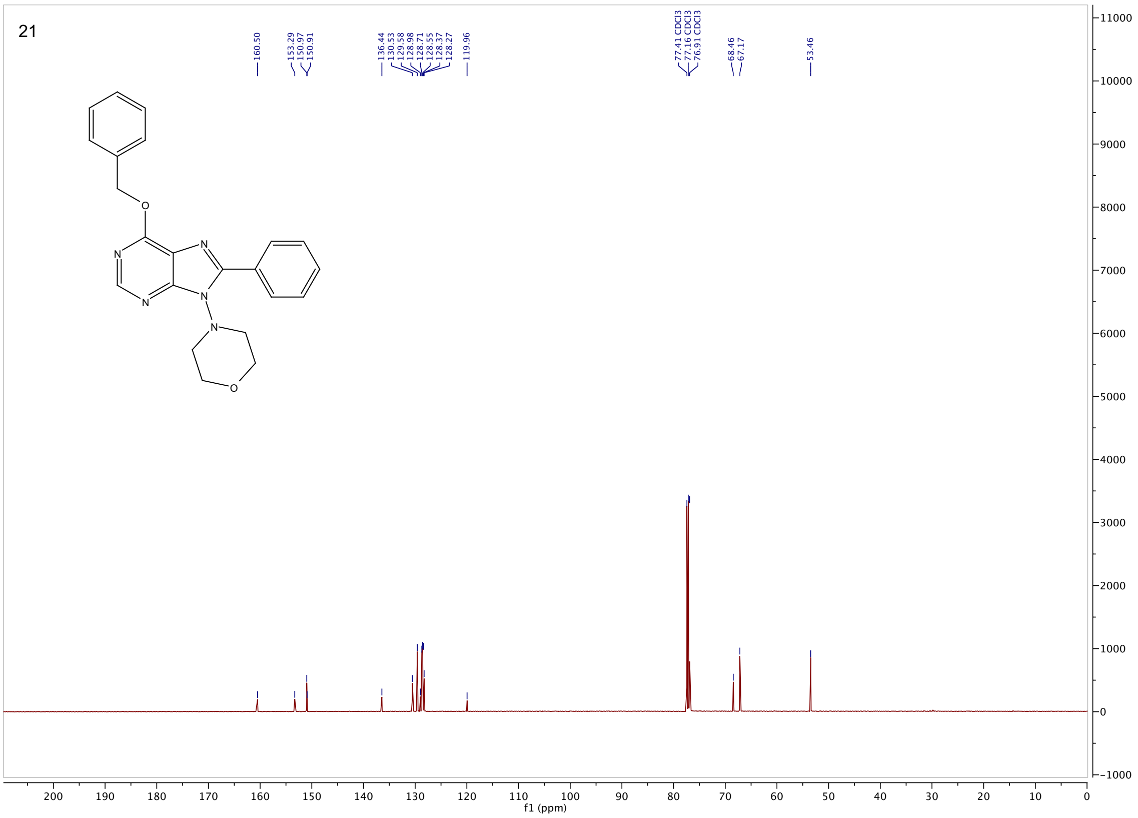

Elemental Composition Report **Page 1**

Single Mass Analysis
Tolerance = 10.0 PPM / DBE: min = -1.5, max = 50.0
Element prediction: Off
Number of isotope peaks used for i-FIT = 3

Monoisotopic Mass, Even Electron Ions
216 formula(e) evaluated with 2 results within limits (up to 50 best isotopic matches for each mass)
Elements Used:
C: 0-38 H: 0-1000 N: 0-8 O: 0-4
ALM-21-1 69 (1.533) AM (Top,6, Ar,5000.0,0.00,1.00)
1: TOF MS ES+

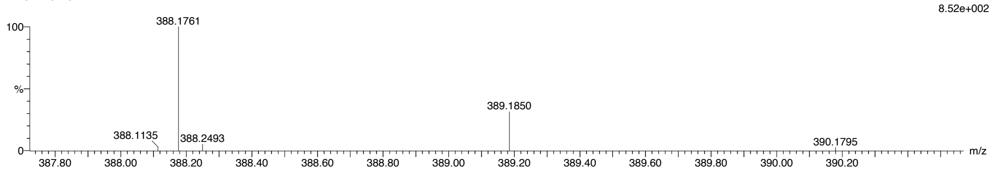

Minimum:				-1.5			
Maximum:		5.0	10.0	50.0			
Mass	Calc. Mass	mDa	PPM	DBE	i-FIT	i-FIT (Norm)	Formula
388.1761	388.1774	-1.3	-3.3	14.5	19.7	0.1	C22 H22 N5 O2
	388.1733	2.8	7.2	10.5	22.1	2.4	C17 H22 N7 O4

4-(6-(Benzyloxy)-8-methyl-9*H*-purin-9-yl)morpholine (**22**). ^{1}H NMR (500 MHz, $CDCl_3$) δ 8.44 (s, 1H), 7.55 – 7.49 (m, 2H), 7.40 – 7.30 (m, 2H), 7.33 – 7.24 (m, 1H), 5.63 (s, 2H), 4.21 (s, 2H), 4.02 – 3.74 (m, 4H), 2.94 (bs, 2H), 2.57 (s, 3H). ^{13}C NMR (126 MHz, $CDCl_3$) δ 159.77, 152.36, 152.14, 150.53, 136.37, 128.60, 128.52, 128.21, 119.12, 68.31, 67.21, 53.65, 13.59. HRMS (ES + ve), $C_{17}H_{20}N_5O_2$ $(M + H)^+$: Calculated 326.1588. Obtained 326.1617.

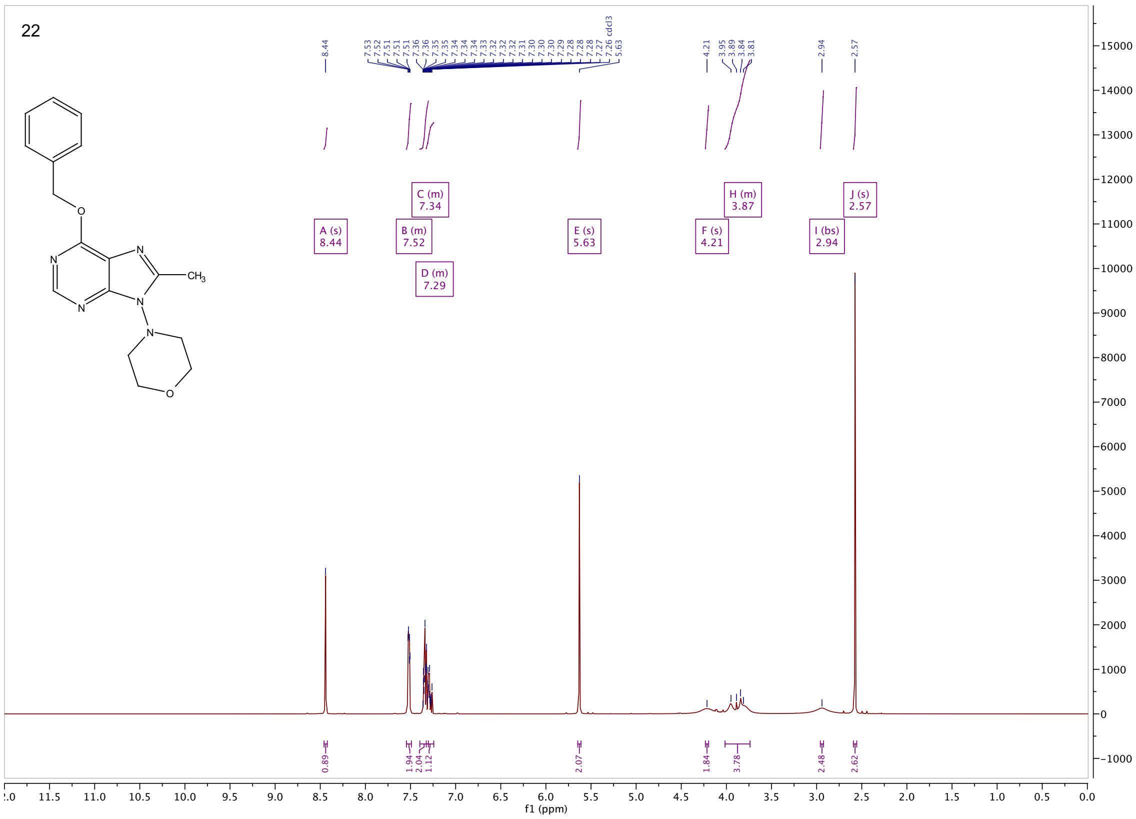

22

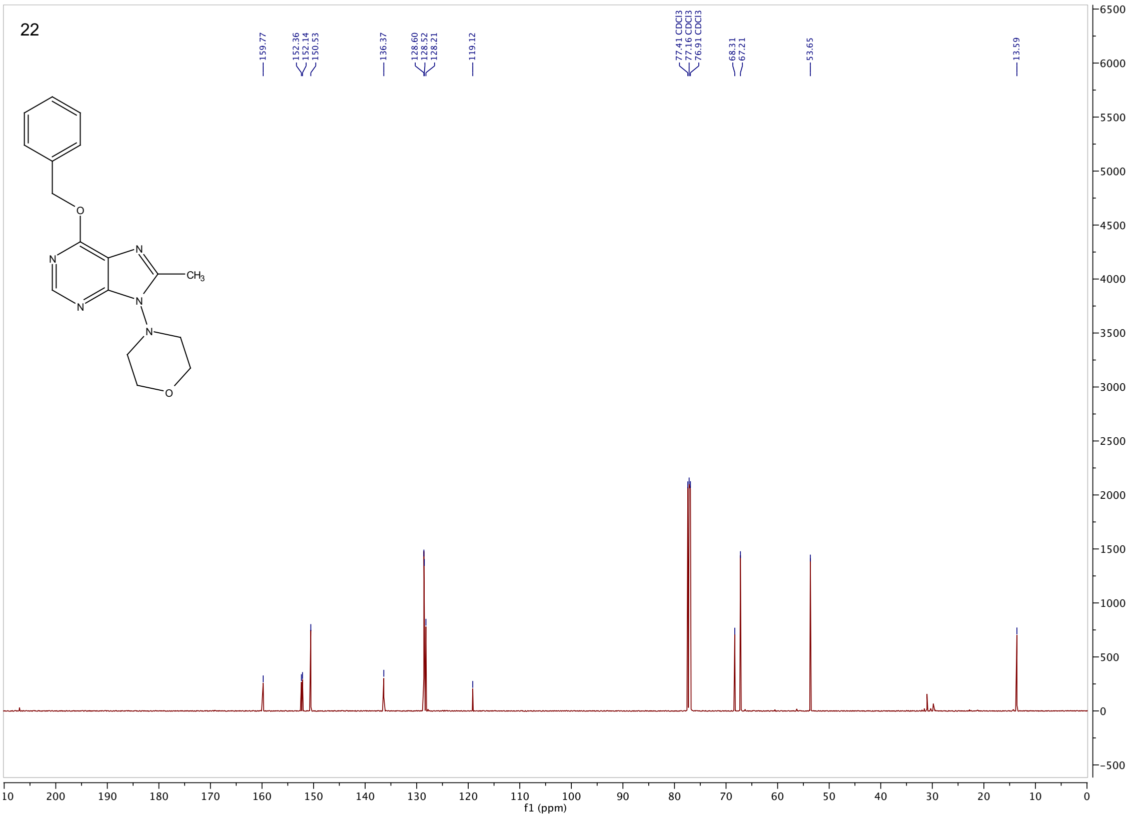

Elemental Composition Report **Page 1**

Single Mass Analysis
Tolerance = 10.0 PPM / DBE: min = -1.5, max = 50.0
Element prediction: Off
Number of isotope peaks used for i-FIT = 3

Monoisotopic Mass, Even Electron Ions
174 formula(e) evaluated with 2 results within limits (up to 50 best isotopic matches for each mass)
Elements Used:
C: 0-23 H: 0-1000 N: 0-8 O: 0-4
ALM-22-1 4 (0.070) AM (Cen,6, 100.00, Ar,5000.0,0.00,1.00)
1: TOF MS ES+

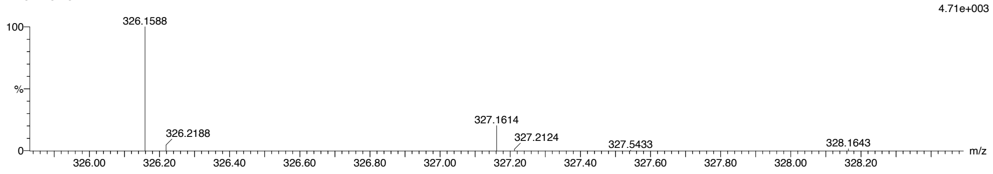

Minimum:				-1.5			
Maximum:		5.0	10.0	50.0			
Mass	Calc. Mass	mDa	PPM	DBE	i-FIT	i-FIT (Norm)	Formula
326.1588	326.1617	-2.9	-8.9	10.5	37.0	2.0	C17 H20 N5 O2
	326.1577	1.1	3.4	6.5	35.1	0.1	C12 H20 N7 O4

4-(6-(Benzyloxy)-9*H*-purin-9-yl)morpholine (**23**). ^{1}H NMR (500 MHz, $CDCl_3$) δ 8.52 (s, 1H), 8.01 (s, 1H), 7.56 – 7.50 (m, 2H), 7.39 – 7.33 (m, 2H), 7.33 – 7.27 (m, 1H), 5.66 (s, 2H), 3.90 (t, *J* = 4.7 Hz, 4H), 3.58 – 3.52 (m, 4H). ^{13}C NMR (126 MHz, $CDCl_3$) δ 160.89, 151.75, 151.18, 141.78, 136.22, 128.56, 128.48, 128.27, 120.72, 68.49, 66.98, 55.00. HRMS (ES + ve), $C_{16}H_{18}N_5O_2$ $(M + H)^+$: Calculated 312.1461. Obtained 312.1434.

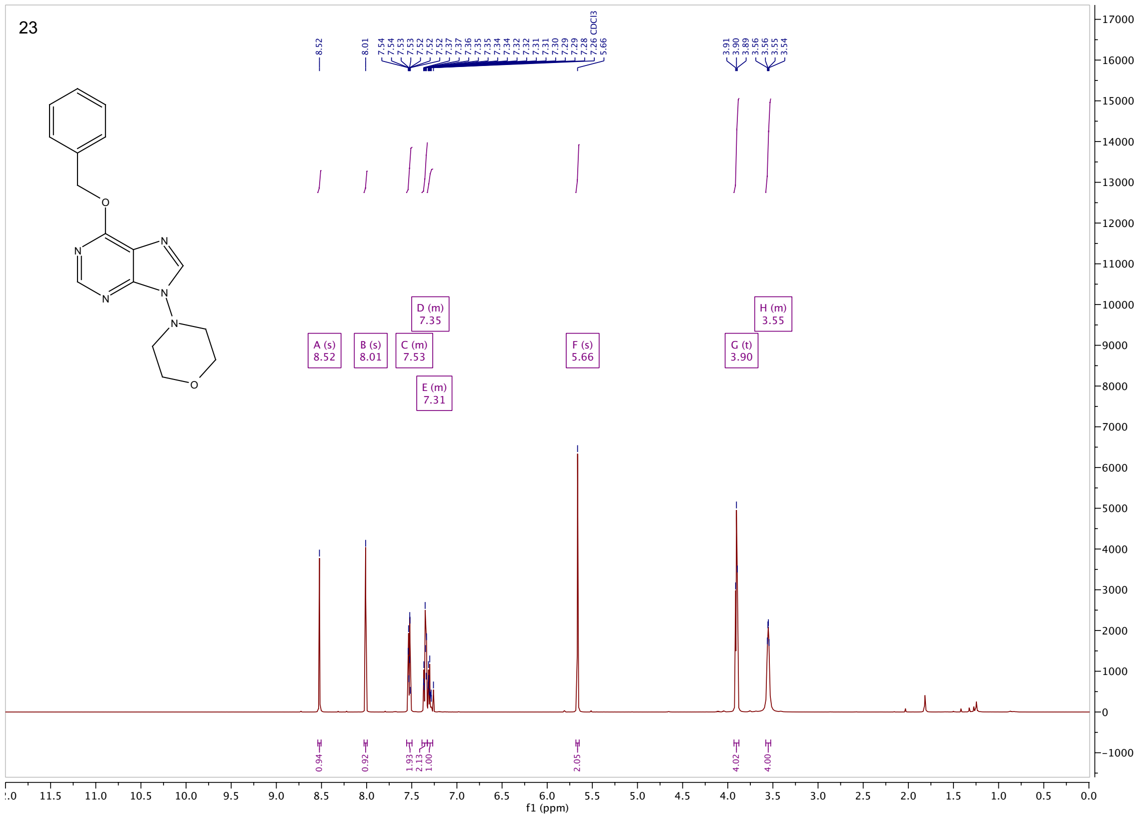

23

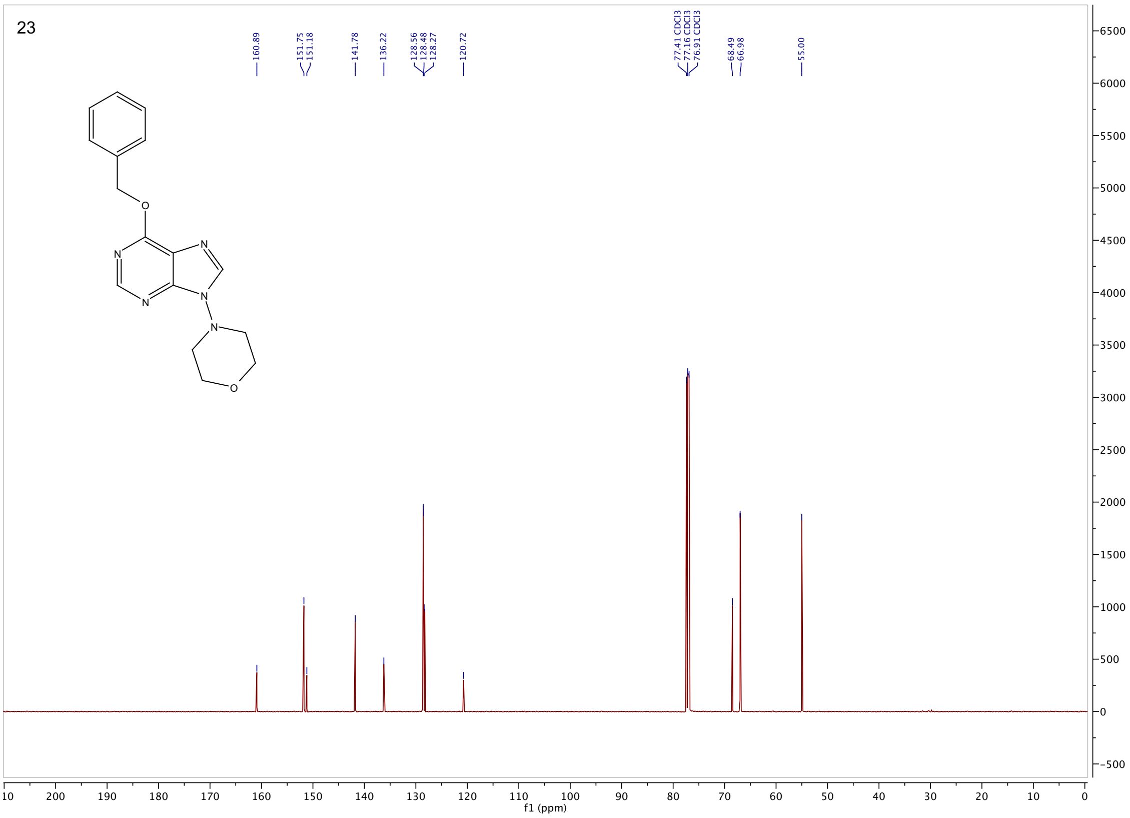

Elemental Composition Report **Page 1**

Single Mass Analysis
Tolerance = 10.0 PPM / DBE: min = -1.5, max = 50.0
Element prediction: Off
Number of isotope peaks used for i-FIT = 3

Monoisotopic Mass, Even Electron Ions
179 formula(e) evaluated with 2 results within limits (up to 50 best isotopic matches for each mass)
Elements Used:
C: 0-38 H: 0-1000 N: 0-8 O: 0-4
ALM-23-1 58 (1.252) AM (Cen,6, 100.00, Ar,5000.0,0.00,1.00)
1: TOF MS ES+

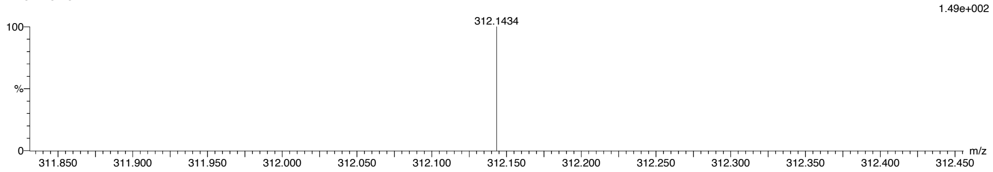

Minimum:				-1.5			
Maximum:		5.0	10.0	50.0			
Mass	Calc. Mass	mDa	PPM	DBE	i-FIT	i-FIT (Norm)	Formula
312.1434	312.1461	-2.7	-8.6	10.5	19.7	1.3	C16 H18 N5 O2
	312.1420	1.4	4.5	6.5	18.7	0.3	C11 H18 N7 O4

6-(Benzyloxy)-9-(4-methylpiperazin-1-yl)-8-phenyl-9*H*-purine (**27**). ^{1}H NMR (500 MHz, $CDCl_3$) δ 8.47 (s, 1H), 8.29 – 8.19 (m, 2H), 7.58 – 7.52 (m, 2H), 7.52 – 7.43 (m, 3H), 7.38 – 7.33 (m, 2H), 7.32 – 7.27 (m, 1H), 5.70 (s, 2H), 4.46 – 4.31 (m, 2H), 3.24 – 2.76 (m, 6H), 2.37 (s, 3H). ^{13}C NMR (126 MHz, $CDCl_3$) δ 160.40, 153.45, 151.02, 150.86, 136.48, 130.42, 129.58, 129.04, 128.68, 128.52, 128.30, 128.22, 119.93, 68.38, 55.20, 52.63, 45.96. HRMS (ES + ve), $C_{23}H_{25}N_6O$ $(M + H)^+$: Calculated 401.2090. Obtained 401.2070.

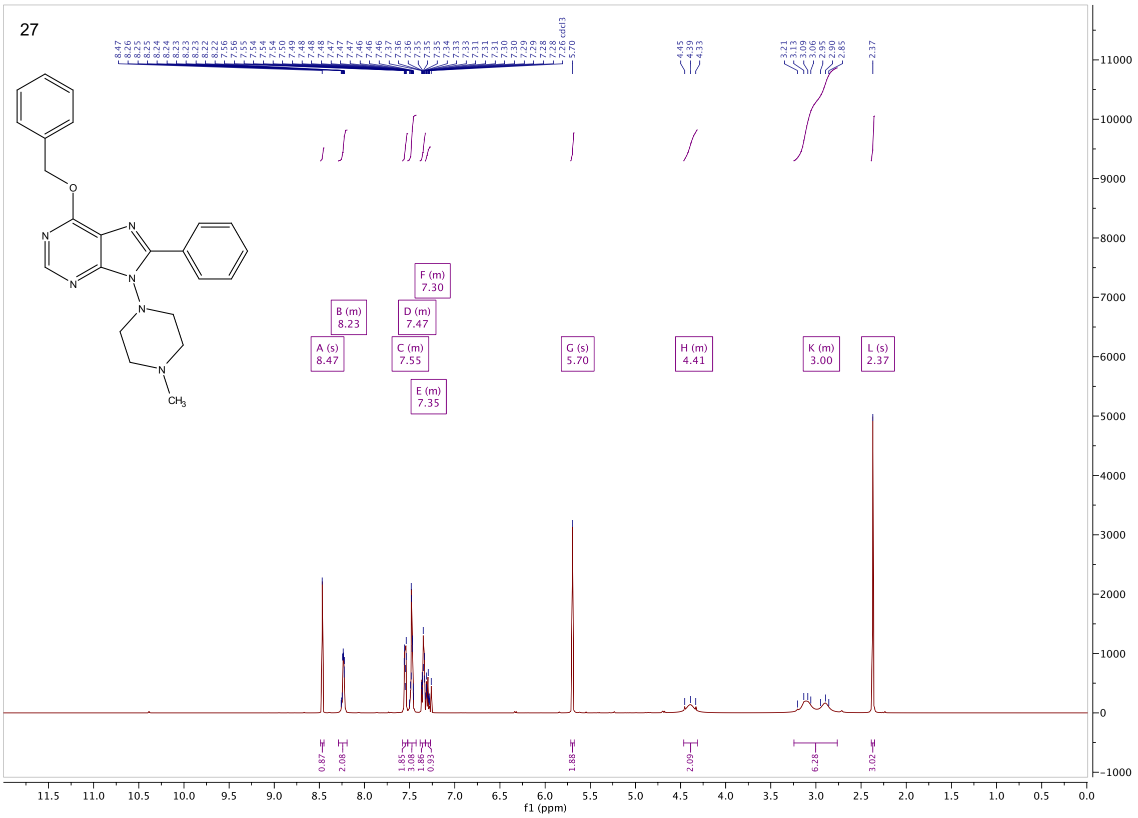

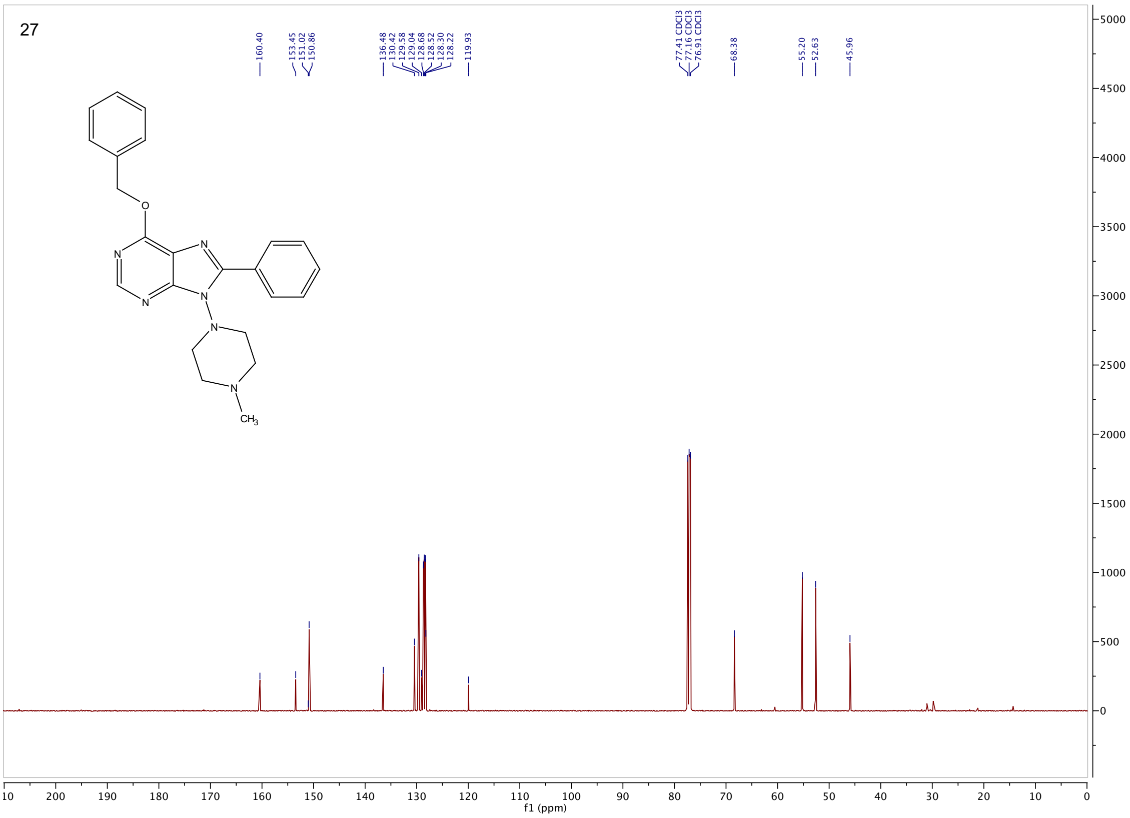

Elemental Composition Report **Page 1**

Single Mass Analysis
Tolerance = 10.0 PPM / DBE: min = -1.5, max = 50.0
Element prediction: Off
Number of isotope peaks used for i-FIT = 3

Monoisotopic Mass, Even Electron Ions
767 formula(e) evaluated with 7 results within limits (up to 50 best isotopic matches for each mass)
Elements Used:
C: 0-23 H: 0-1000 N: 0-8 O: 0-4 S: 0-4
ALM-27-1 13 (0.283) AM (Cen,6, 100.00, Ar,5000.0,0.00,1.00)
1: TOF MS ES+

7.96e+003

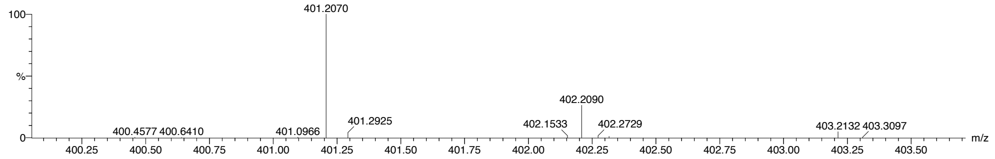

Minimum:				-1.5			
Maximum:		5.0	10.0	50.0			

Mass	Calc. Mass	mDa	PPM	DBE	i-FIT	i-FIT (Norm)	Formula
401.2070	401.2090	-2.0	-5.0	14.5	55.4	0.1	C23 H25 N6 O
	401.2050	2.0	5.0	10.5	57.2	2.0	C18 H25 N8 O3
	401.2083	-1.3	-3.2	5.5	63.7	8.5	C15 H29 N8 O3 S
	401.2085	-1.5	-3.7	8.5	67.1	11.9	C23 H33 N2 S2
	401.2045	2.5	6.2	4.5	67.3	12.1	C18 H33 N4 O2 S2
	401.2079	-0.9	-2.2	-0.5	69.1	13.9	C15 H37 N4 O2 S3
	401.2040	3.0	7.5	-1.5	70.4	15.2	C18 H41 O S4

6-(Benzyloxy)-8-methyl-9-(4-methylpiperazin-1-yl)-9*H*-purine (**28**). ^{1}H NMR (500 MHz, $CDCl_3$) δ 8.41 (s, 1H), 7.51 (d, J = 7.0 Hz, 2H), 7.37 – 7.31 (m, 2H), 7.31 – 7.26 (m, 1H), 5.62 (s, 2H), 4.29 – 4.13 (m, 2H), 3.09 – 2.75 (m, 6H), 2.55 (s, 3H), 2.36 (s, 3H). ^{13}C NMR (126 MHz, $CDCl_3$) δ 159.67, 152.52, 152.28, 150.43, 136.44, 128.58, 128.49, 128.16, 119.11, 68.23, 55.25, 52.87, 45.95, 13.56. HRMS (ES + ve), $C_{18}H_{23}N_6O$ $(M + H)^+$: Calculated 339.1933. Obtained 339.1955.

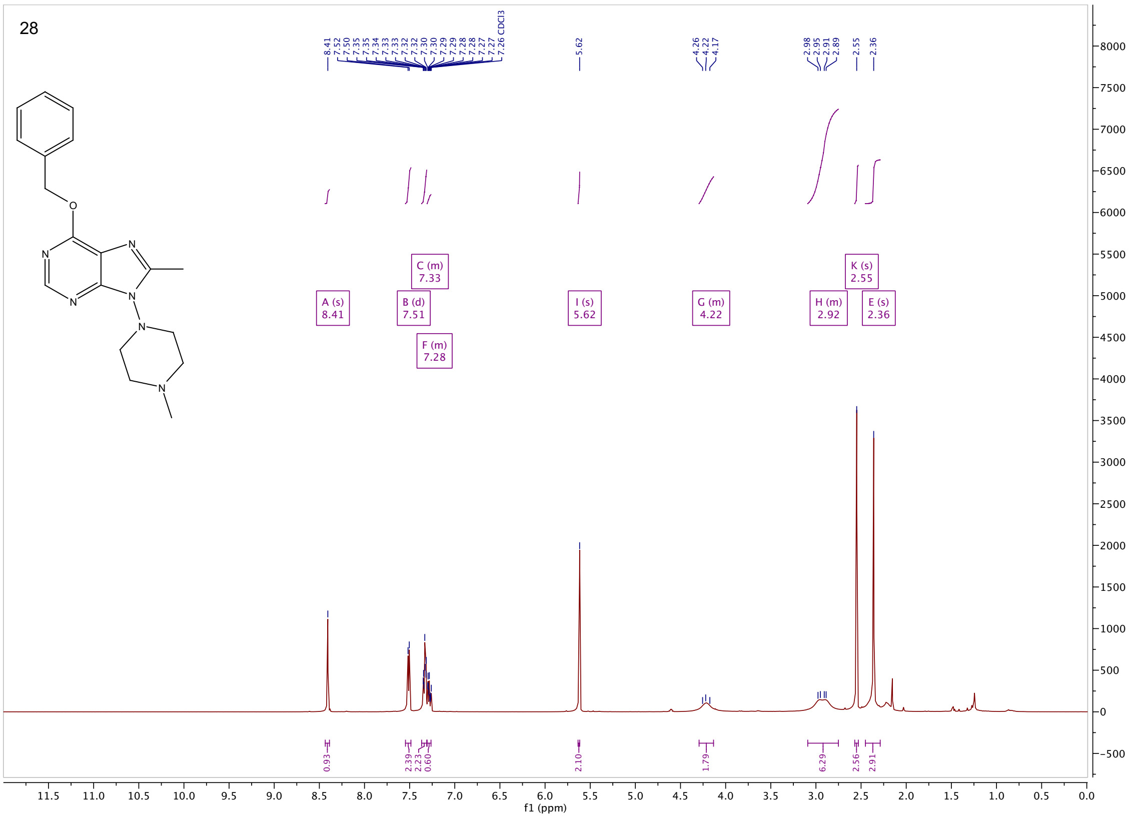

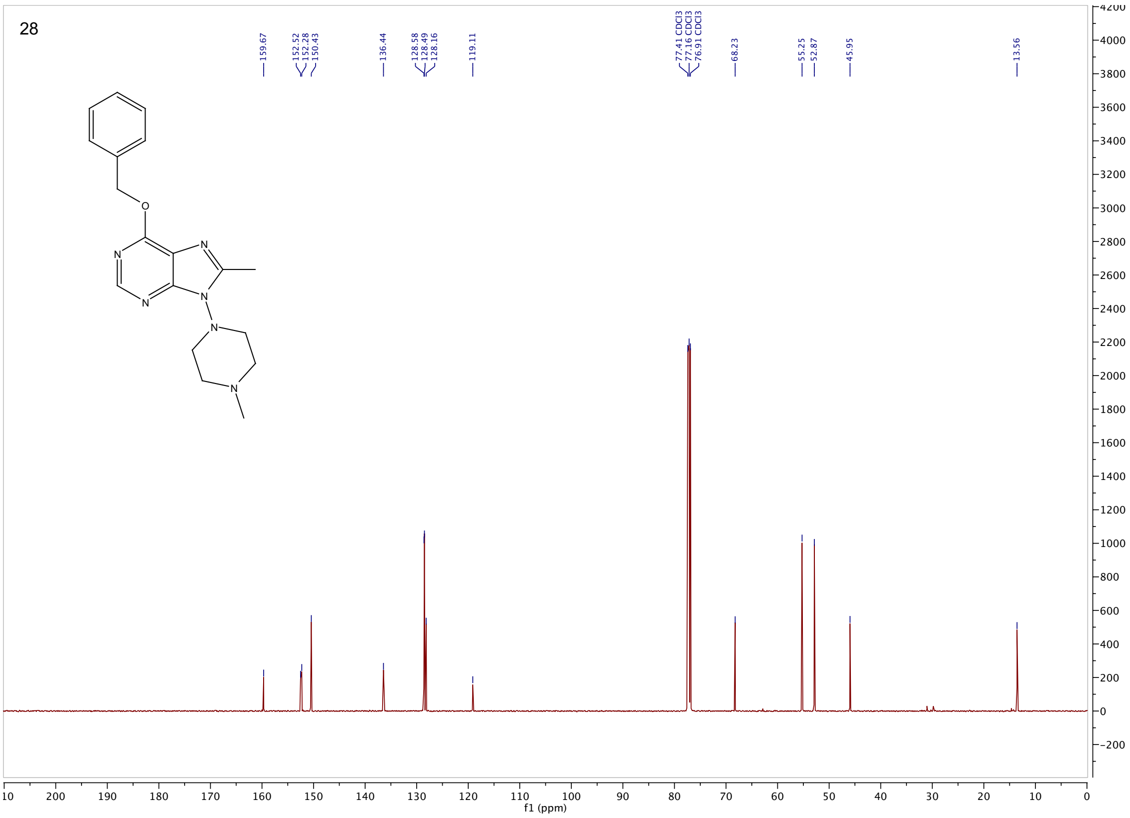

Elemental Composition Report **Page 1**

Single Mass Analysis
Tolerance = 10.0 PPM / DBE: min = -1.5, max = 50.0
Element prediction: Off
Number of isotope peaks used for i-FIT = 3

Monoisotopic Mass, Even Electron Ions
103 formula(e) evaluated with 1 results within limits (up to 50 best isotopic matches for each mass)
Elements Used:
C: 0-18 H: 0-1000 N: 0-8 O: 0-4
ALM-28-1 142 (3.103) AM (Top,6, Ar,5000.0,0.00,1.00)
1: TOF MS ES+

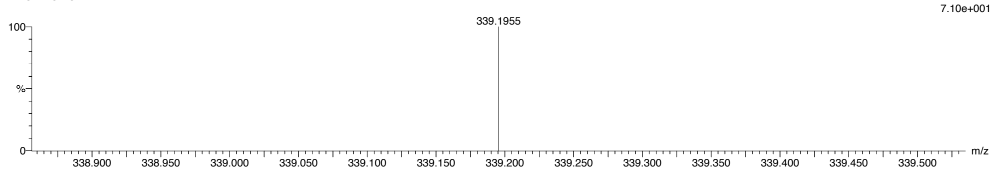

Minimum:				-1.5			
Maximum:		5.0	10.0	50.0			
Mass	Calc. Mass	mDa	PPM	DBE	i-FIT	i-FIT (Norm)	Formula
339.1955	339.1933	2.2	6.5	10.5	17.6	0.0	C18 H23 N6 O

6-(Benzyloxy)-9-(4-methylpiperazin-1-yl)-9*H*-purine (**29**). ^{1}H NMR (500 MHz, $CDCl_3$) δ 8.51 (s, 1H), 8.03 (s, 1H), 7.56 – 7.50 (m, 2H), 7.40 – 7.27 (m, 3H), 5.66 (s, 2H), 3.57 – 3.53 (m, 4H), 2.69 – 2.65 (m, 4H), 2.37 (s, 3H). ^{13}C NMR (126 MHz, $CDCl_3$) δ 160.85, 151.80, 151.24, 141.55, 136.28, 128.57, 128.48, 128.26, 120.58, 68.47, 54.97, 54.52, 45.87. HRMS (ES + ve), $C_{17}H_{21}N_6O$ $(M + H)^+$: Calculated 325.177. Obtained 325.1750.

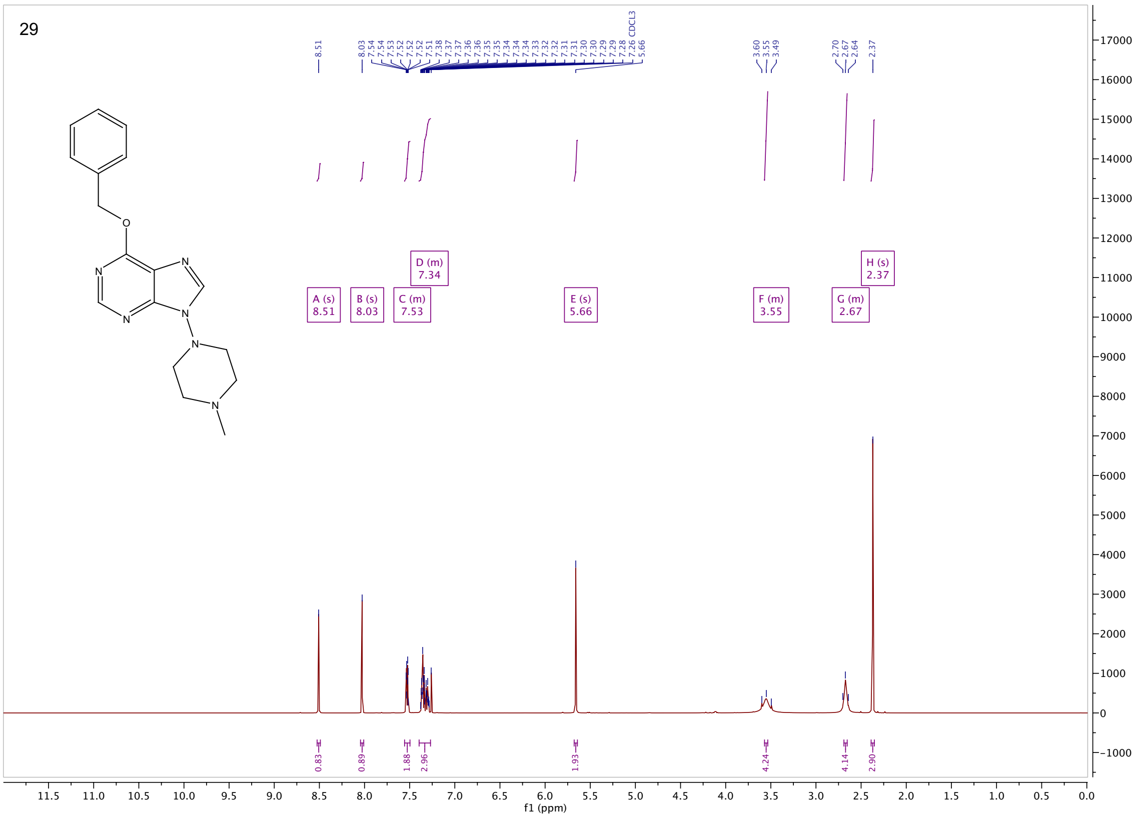

29

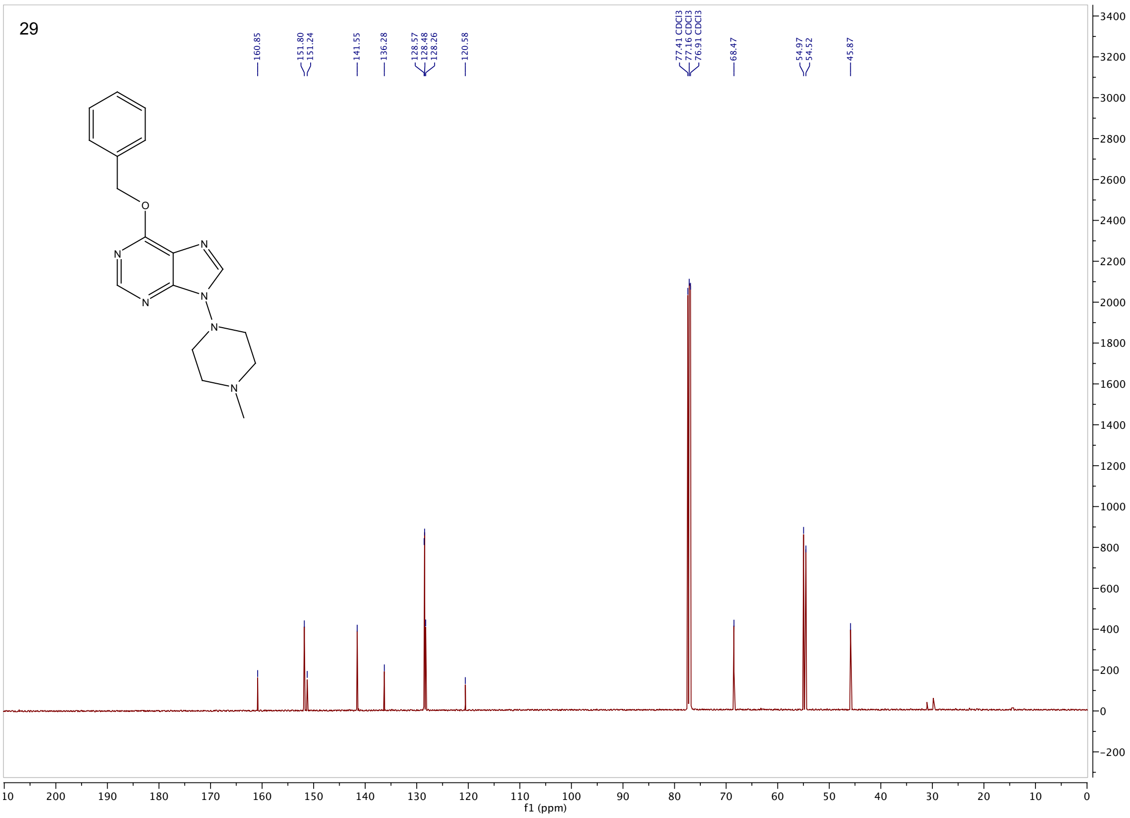

Elemental Composition Report **Page 1**

Single Mass Analysis
Tolerance = 10.0 PPM / DBE: min = -1.5, max = 50.0
Element prediction: Off
Number of isotope peaks used for i-FIT = 3

Monoisotopic Mass, Even Electron Ions
116 formula(e) evaluated with 2 results within limits (up to 50 best isotopic matches for each mass)
Elements Used:
C: 0-18 H: 0-1000 N: 0-8 O: 0-4
ALM-29-1 11 (0.246) AM (Cen,6, 100.00, Ar,5000.0,0.00,1.00)
1: TOF MS ES+

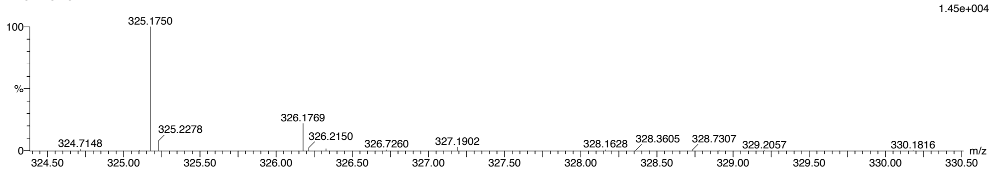

	Calc. Mass	mDa	PPM	DBE	i-FIT	i-FIT (Norm)	Formula
Minimum:				-1.5			
Maximum:		5.0	10.0	50.0			

Mass	Calc. Mass	mDa	PPM	DBE	i-FIT	i-FIT (Norm)	Formula
325.1750	325.1777	-2.7	-8.3	10.5	60.4	0.0	C17 H21 N6 O
	325.1737	1.3	4.0	6.5	66.7	6.3	C12 H21 N8 O3

6-Ethoxy-9-isopropyl-9*H*-purine (**MJ-1**). ^{1}H NMR (500 MHz, Chloroform-*d*) δ 8.48 (s, 1H), 7.94 (s, 1H), 4.90 – 4.81 (m, 1H), 4.67 – 4.59 (m, 2H), 1.62 – 1.57 (m, 6H), 1.51 – 1.44 (m, 3H). ^{13}C NMR (126 MHz, Chloroform-*d*) δ 160.93, 151.83, 139.66, 121.95, 63.09, 47.45, 22.71, 14.62. HRMS (ES + ve), $C_{10}H_{15}N_4O$ $(M + H)^+$: Calculated 207.1246. Obtained 207.1245.

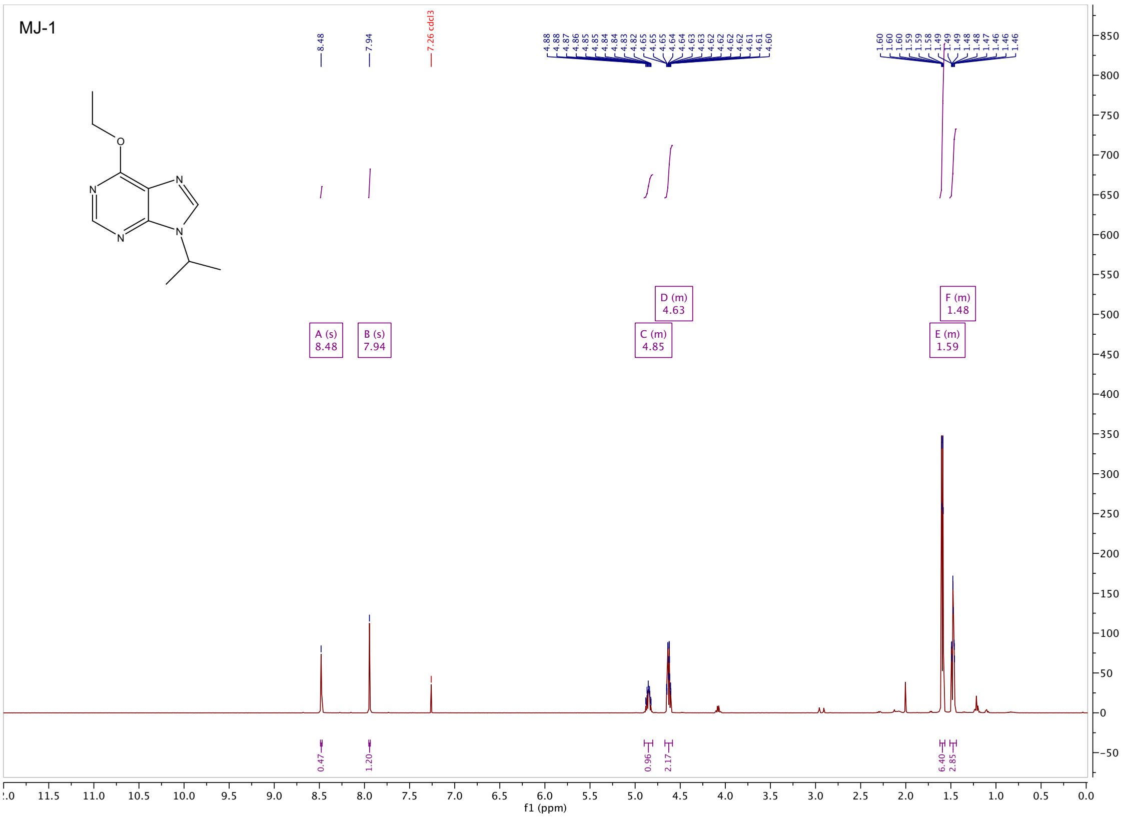

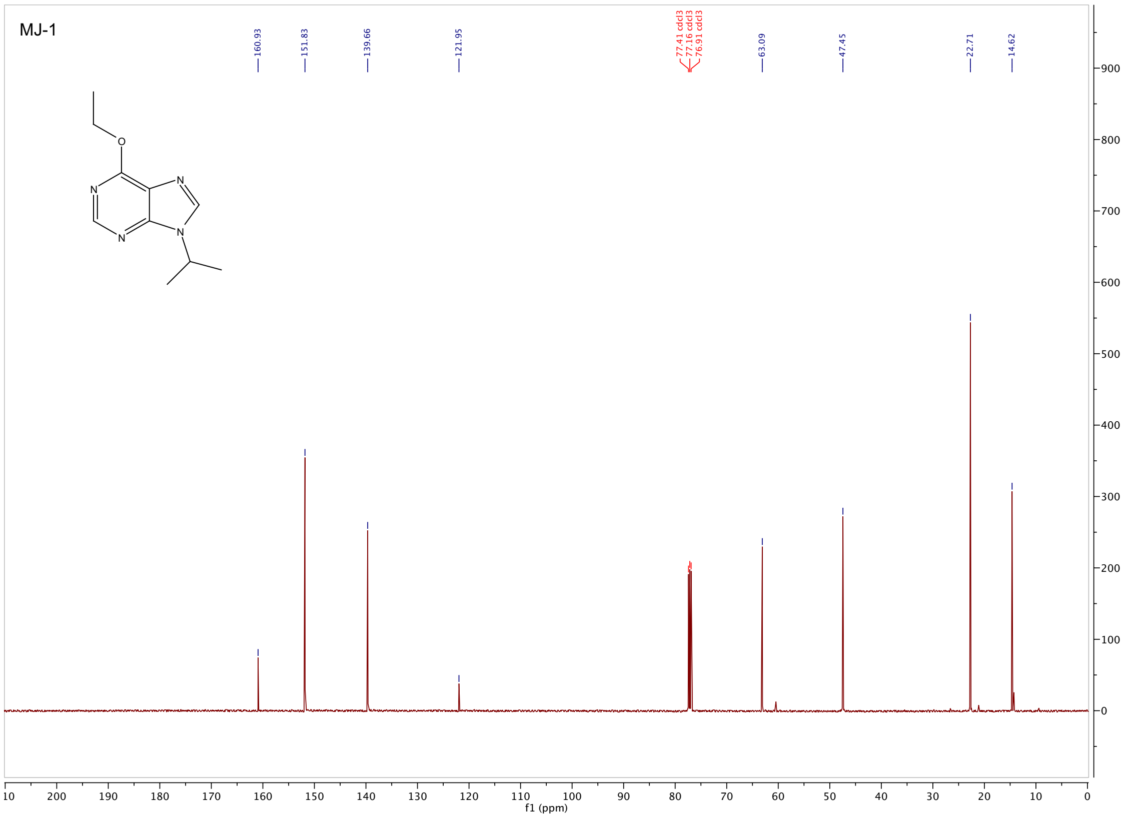

Elemental Composition Report **Page 1**

Single Mass Analysis
Tolerance = 5.0 PPM / DBE: min = -1.5, max = 50.0
Element prediction: Off
Number of isotope peaks used for i-FIT = 3

Monoisotopic Mass, Even Electron Ions
369 formula(e) evaluated with 1 results within limits (up to 50 best isotopic matches for each mass)
Elements Used:
C: 0-22 H: 0-1000 N: 0-5 O: 0-6 F: 0-3

12/3289

ASIMJ-1 40 (0.885) 1: TOF MS ES+ 1.90e+002

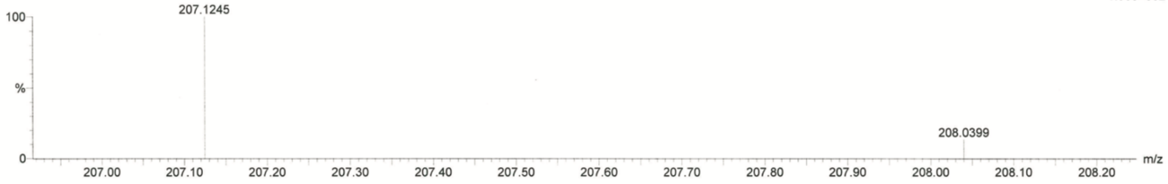

Minimum:				-1.5		
Maximum:		20.0	5.0	50.0		
Mass	Calc. Mass	mDa	PPM	DBE	i-FIT	Formula
207.1245	207.1246	-0.1	-0.5	5.5	n/a	C10 H15 N4 O

6-(Benzyloxy)-9-isopropyl-8-methyl-9*H*-purine (**MJ-5**). ^{1}H NMR (500 MHz, $CDCl_3$) δ 8.46 (s, 1H), 7.57 – 7.48 (m, 2H), 7.39 – 7.30 (m, 2H), 7.33 – 7.26 (m, 1H), 5.64 (s, 2H), 4.80 – 4.68 (m, 1H), 2.63 (s, 3H), 1.68 (d, $J = 6.8$ Hz, 6H). ^{13}C NMR (126 MHz, $CDCl_3$) δ 159.58, 153.54, 150.86, 150.57, 136.59, 128.60, 128.52, 128.15, 120.90, 68.23, 48.52, 21.40, 15.33. HRMS (ES + ve), $C_{16}H_{19}N_4O$ $(M + H)^+$: Calculated 283.1559. Obtained 283.1558.

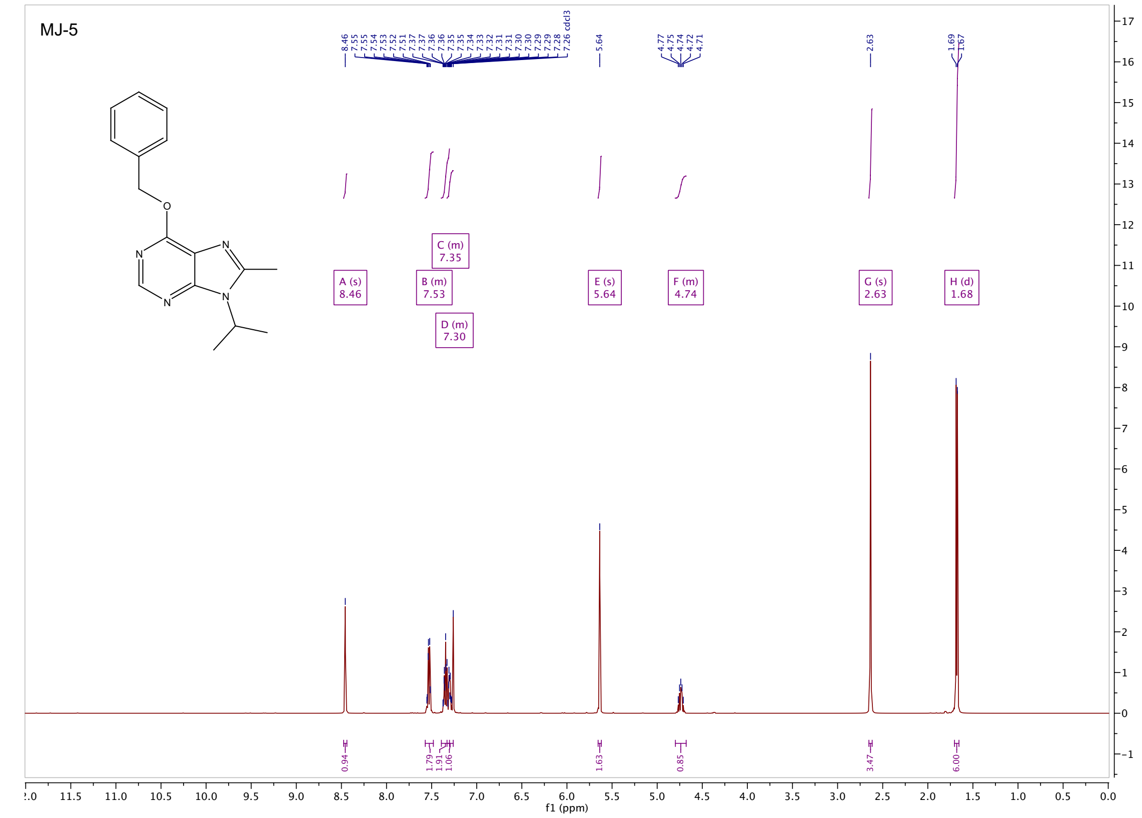

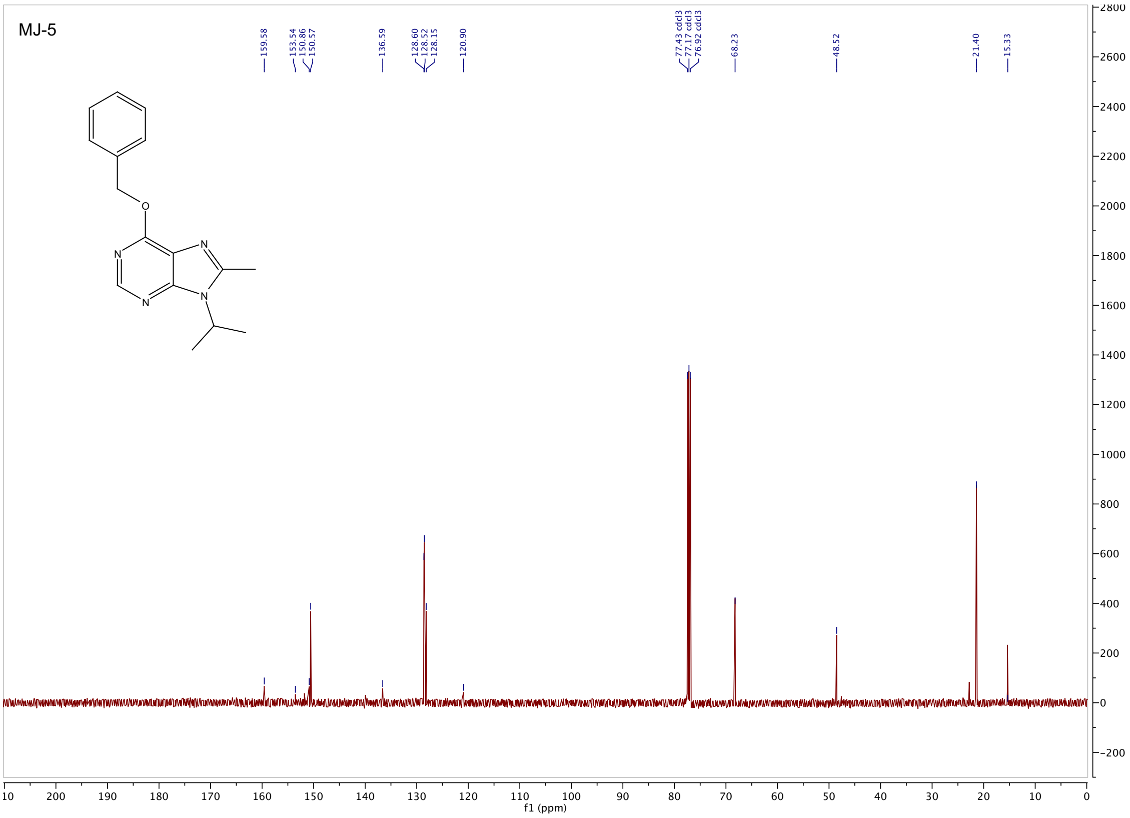

Elemental Composition Report — **Page 1**

Single Mass Analysis
Tolerance = 5.0 PPM / DBE: min = -1.5, max = 50.0
Element prediction: Off
Number of isotope peaks used for i-FIT = 3

Monoisotopic Mass, Even Electron Ions
525 formula(e) evaluated with 4 results within limits (up to 50 best isotopic matches for each mass)
Elements Used:
C: 0-22 H: 0-1000 N: 0-5 O: 0-6 F: 0-3

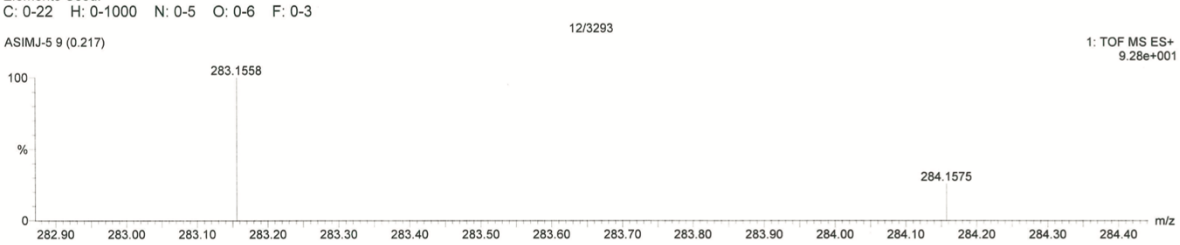

Mass	Calc. Mass	mDa	PPM	DBE	i-FIT	Formula
Minimum:				-1.5		
Maximum:		20.0	5.0	50.0		
283.1558	283.1559	-0.1	-0.4	9.5	n/a	C16 H19 N4 O
	283.1570	-1.2	-4.2	5.5	n/a	C13 H20 N4 O2 F
	283.1545	1.3	4.6	4.5	n/a	C15 H23 O5
	283.1557	0.1	0.4	0.5	n/a	C12 H24 O6 F

9-Benzyl-6-ethoxy-9*H*-purine (**MJ-7**). ^{1}H NMR (500 MHz, $CDCl_3$) δ 8.51 (s, 1H), 7.84 (s, 1H), 7.33 – 7.26 (m, 3H), 7.24 (dd, J = 8.0, 2.3 Hz, 2H), 5.37 (s, 2H), 4.63 (q, J = 7.1 Hz, 2H), 1.47 (t, J = 7.1 Hz, 3H). ^{13}C NMR (126 MHz, $CDCl_3$) δ 161.07, 152.47, 152.33, 141.95, 135.48, 129.22, 128.62, 127.88, 121.53, 63.29, 47.56, 14.67. HRMS (ES + ve), $C_{14}H_{15}N_4O$ $(M + H)^+$: Calculated 255.1246. Obtained 255.1252.

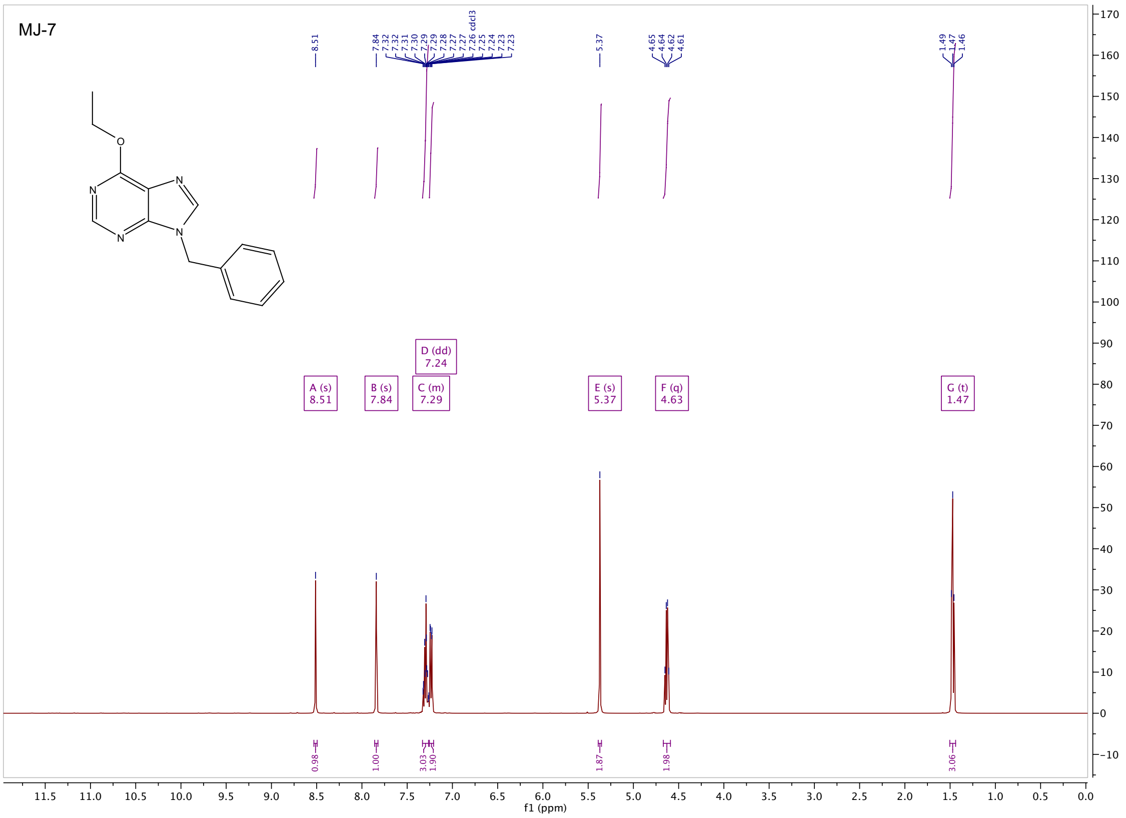

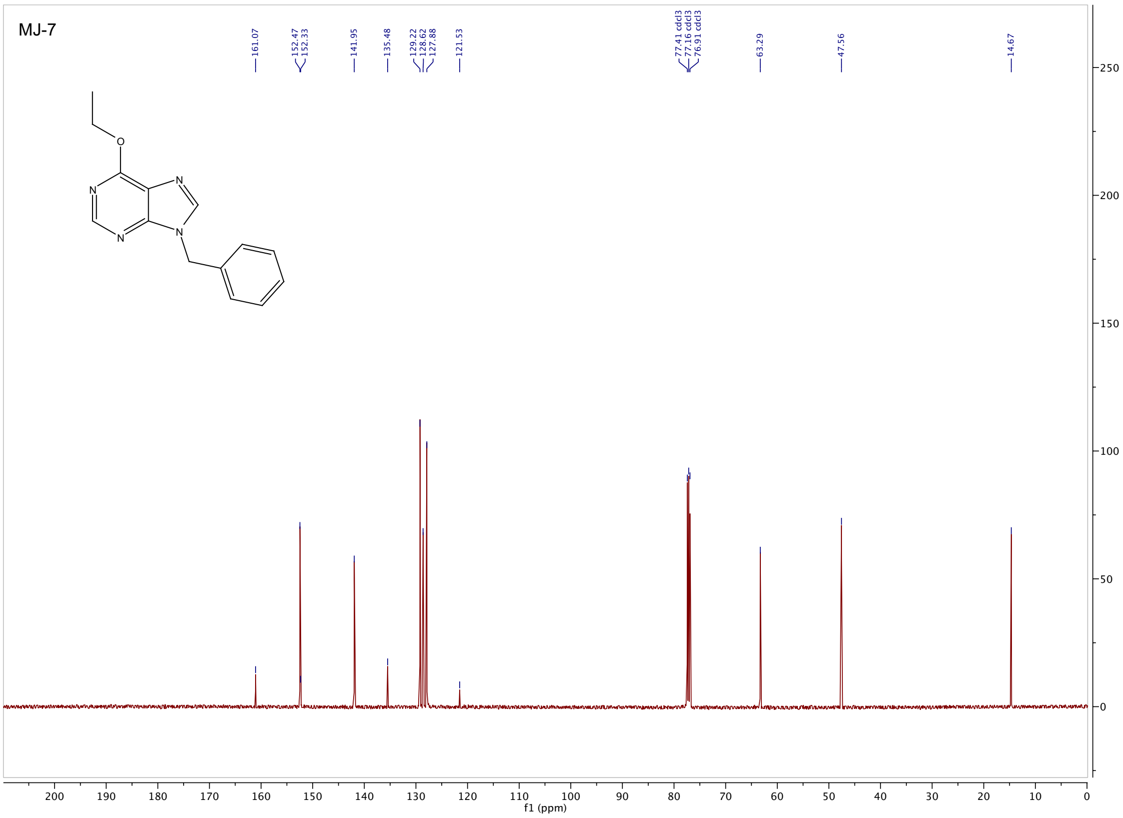

5f. - 9-Benzyl-6-ethoxy-9H-purine (MS)

Elemental Composition Report

Page 1

Single Mass Analysis

Tolerance = 5.0 PPM / DBE: min = -1.5, max = 50.0
Element prediction: Off
Number of isotope peaks used for i-FIT = 3

Monoisotopic Mass, Even Electron Ions
1456 formula(e) evaluated with 4 results within limits (up to 50 best isotopic matches for each mass)
Elements Used:
C: 0-16 H: 0-1000 N: 0-5 O: 0-6 Na: 0-1 S: 0-1 Cl: 0-4

7 173 (3.827)

1: TOF MS ES+
8.63e+001

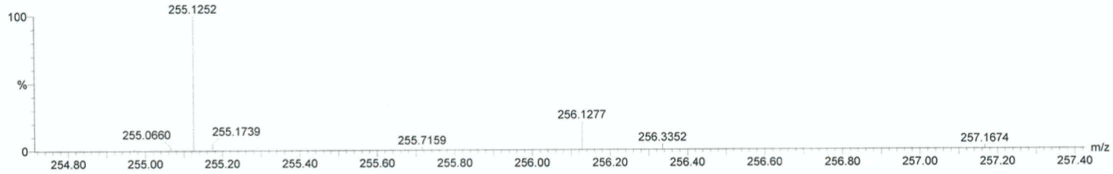

Minimum:				-1.5		
Maximum:		20.0	5.0	50.0		
Mass	Calc. Mass	mDa	PPM	DBE	i-FIT	Formula
255.1252	255.1246	0.6	2.4	9.5	n/a	C14 H15 N4 O
	255.1256	-0.4	-1.6	1.5	n/a	C9 H20 N4 O Na S
	255.1264	-1.2	-4.7	4.5	n/a	C13 H20 N2 O Cl
	255.1240	1.2	4.7	1.5	n/a	C11 H21 N2 O Na Cl

9-Benzyl-6-ethoxy-8-methyl-9*H*-purine (**MJ-8**). ^{1}H NMR (500 MHz, $CDCl_3$) δ 8.50 (s, 1H), 7.35 – 7.26 (m, 2H), 7.17 – 7.10 (m, 2H), 5.40 (s, 2H), 4.65 (q, J = 7.1 Hz, 2H), 2.51 (s, 3H), 1.51 (t, J = 7.1 Hz, 3H). ^{13}C NMR (126 MHz, $CDCl_3$) δ 159.98, 153.69, 151.66, 151.57, 135.65, 129.13, 128.25, 127.07, 120.49, 63.10, 46.23, 14.73, 14.54. HRMS (ES + ve), $C_{15}H_{17}N_4O$ $(M + H)^+$: Calculated 269.1402. Obtained 269.1396.

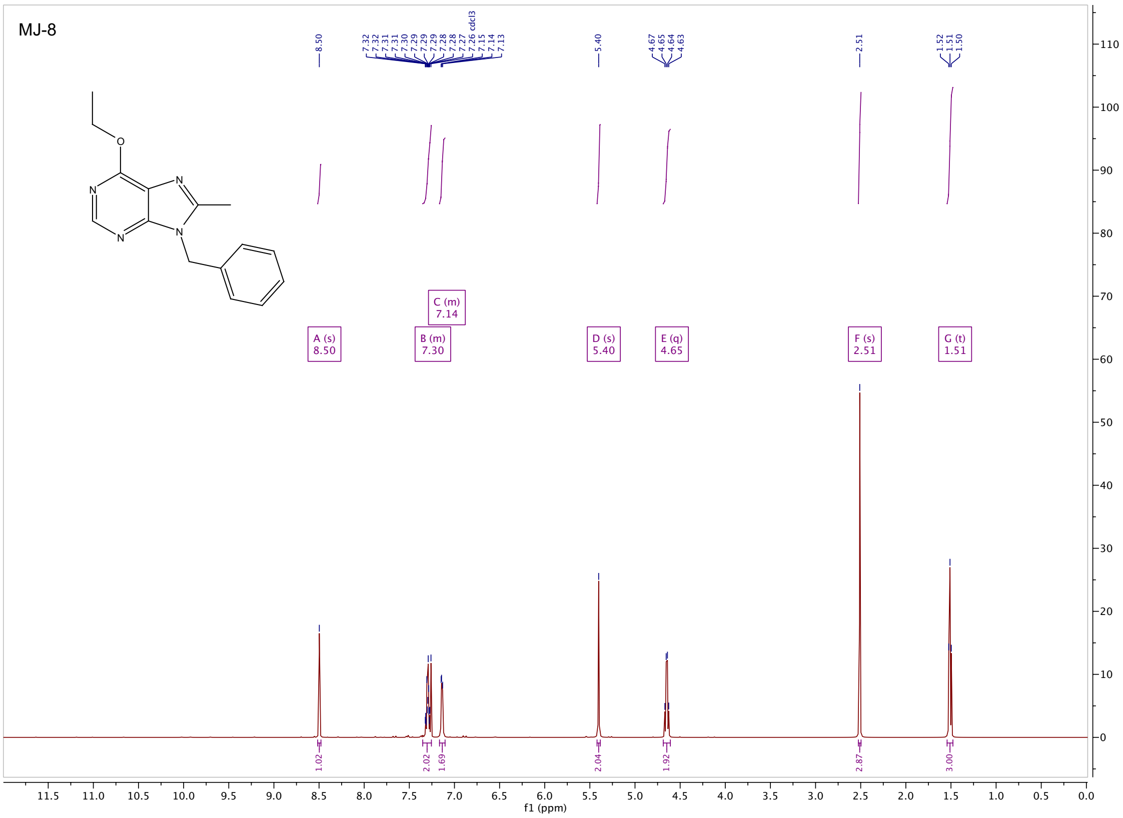

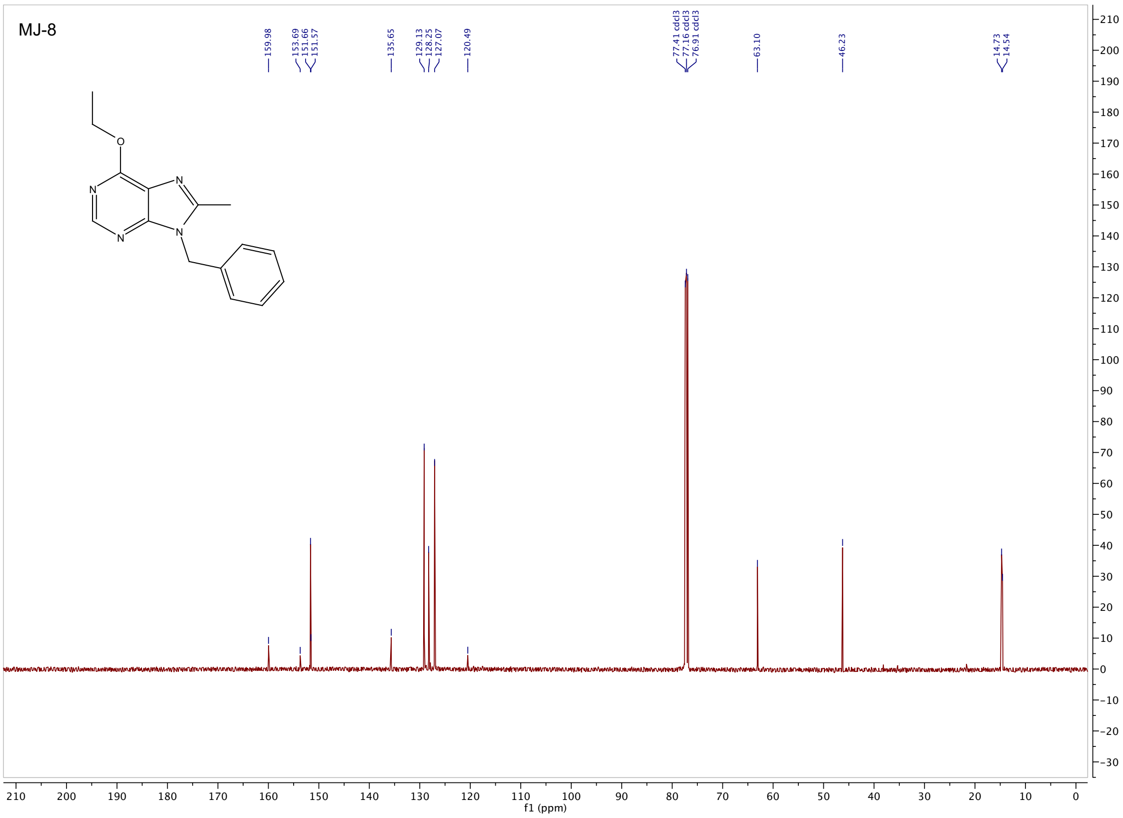

5g.- 9-Benzyl-6-ethoxy-8-methyl-9H-purine (MS)

Elemental Composition Report

Page 1

Single Mass Analysis

Tolerance = 5.0 PPM / DBE: min = -1.5, max = 50.0
Element prediction: Off
Number of isotope peaks used for i-FIT = 3

Monoisotopic Mass, Even Electron Ions
1585 formula(e) evaluated with 3 results within limits (up to 50 best isotopic matches for each mass)
Elements Used:
C: 0-16 H: 0-1000 N: 0-5 O: 0-6 Na: 0-1 S: 0-1 Cl: 0-4

8 9 (0.212)

1: TOF MS ES+
6.73e+002

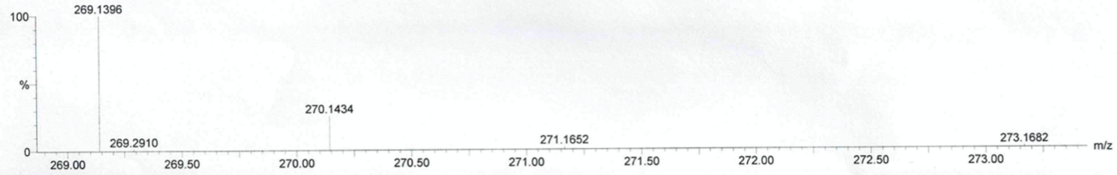

Mass	Calc. Mass	mDa	PPM	DBE	i-FIT	Formula
Minimum:				-1.5		
Maximum:		20.0	5.0	50.0		
269.1396	269.1402	-0.6	-2.2	9.5	6.0	C15 H17 N4 O
	269.1389	0.7	2.6	4.5	11.4	C14 H21 O5
	269.1397	-0.1	-0.4	1.5	n/a	C12 H23 N2 O Na Cl

9-Benzyl-6-(benzyloxy)-9*H*-purine (**MJ-11**). ^{1}H NMR (500 MHz, $CDCl_3$) δ 8.58 (s, 1H), 7.89 (s, 1H), 7.58 – 7.51 (m, 2H), 7.40 – 7.25 (m, 8H), 5.69 (s, 2H), 5.41 (s, 2H). ^{13}C NMR (126 MHz, $CDCl_3$) δ 160.77, 152.36, 142.17, 136.33, 135.44, 129.25, 128.66, 128.57, 128.50, 128.25, 127.92, 127.11, 121.59, 68.58, 47.62. HRMS (ES + ve), $C_{19}H_{17}N_4O$ $(M + H)^+$: Calculated 317.1402. Obtained 317.1407.

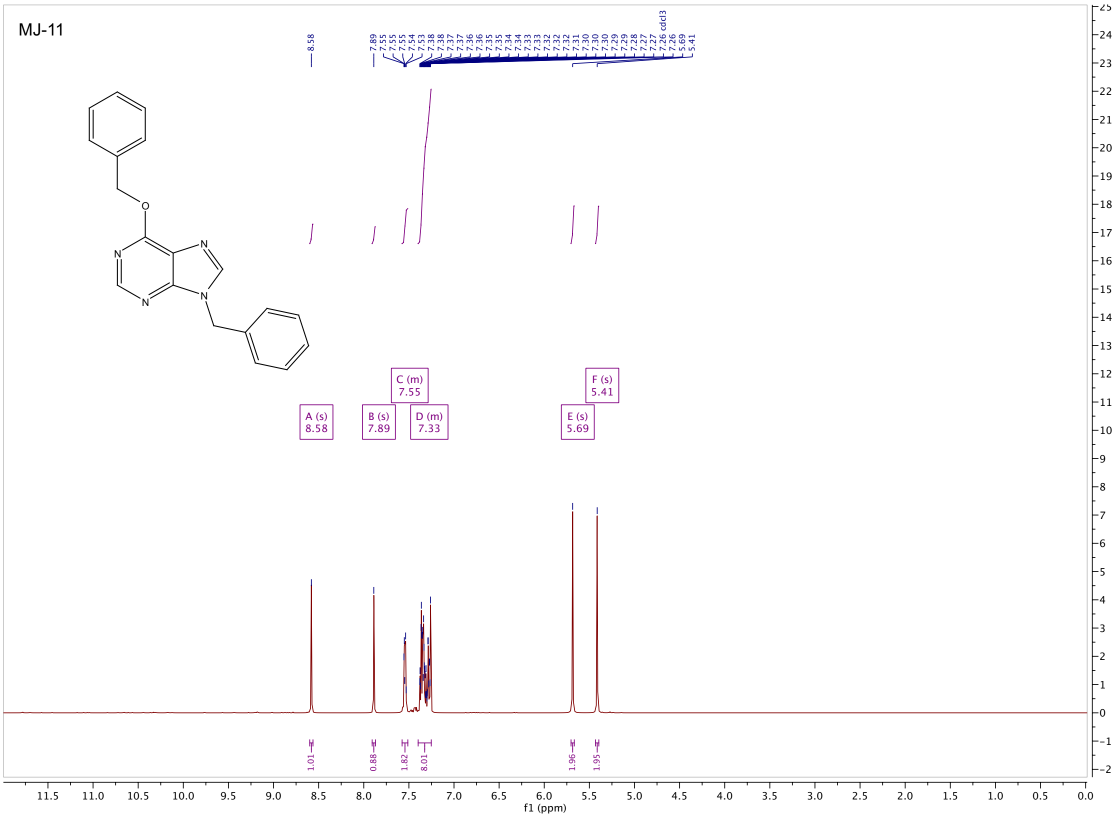

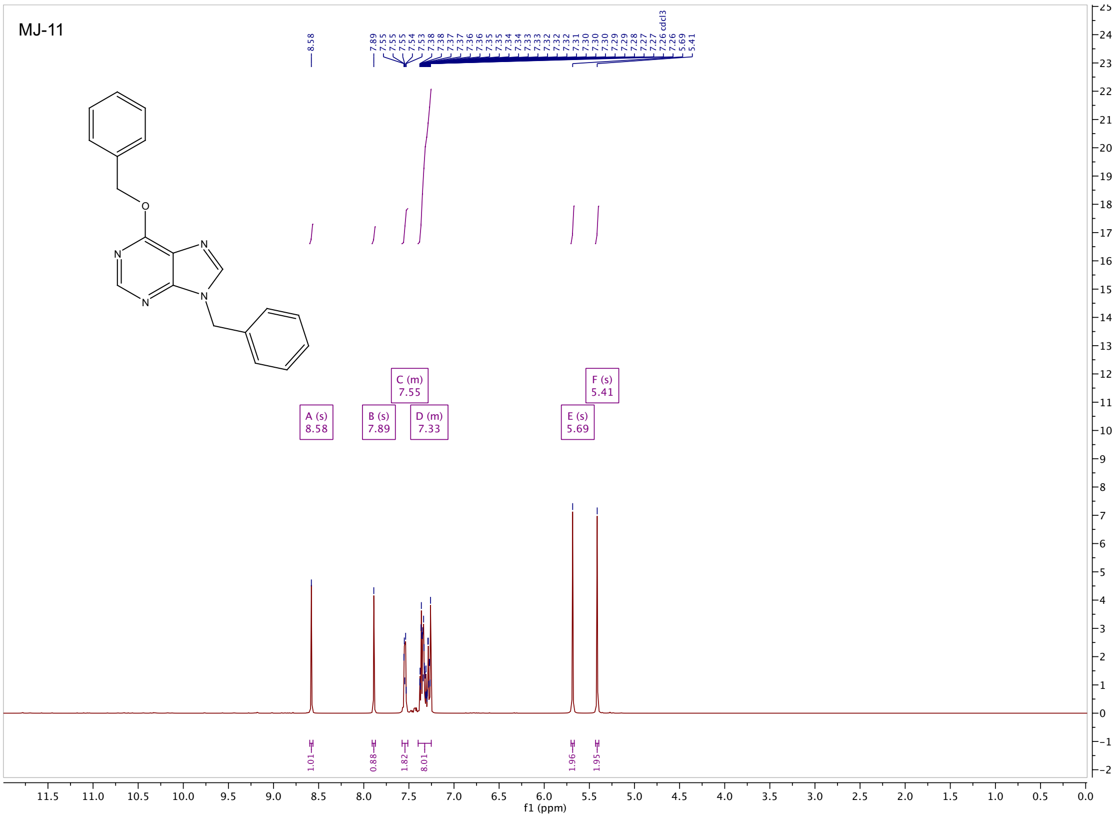

5i- 9-Benzyl-6-(benzyloxy)-9H-purine (MS)

Elemental Composition Report **Page 1**

Single Mass Analysis
Tolerance = 5.0 PPM / DBE: min = -1.5, max = 50.0
Element prediction: Off
Number of isotope peaks used for i-FIT = 3

Monoisotopic Mass, Even Electron Ions
573 formula(e) evaluated with 3 results within limits (up to 50 best isotopic matches for each mass)
Elements Used:
C: 0-22 H: 0-1000 N: 0-5 O: 0-6 F: 0-3

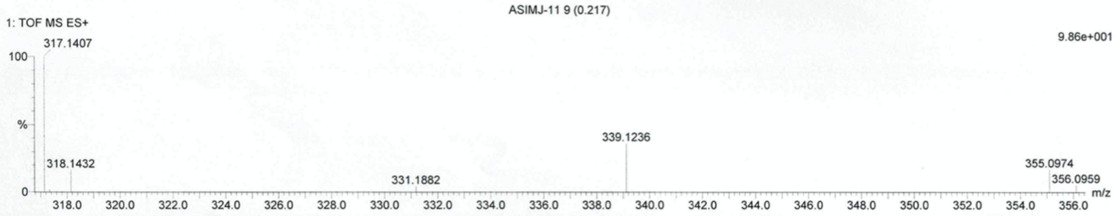

Mass	Calc. Mass	mDa	PPM	DBE	i-FIT	Formula
Minimum:				-1.5		
Maximum:		20.0	5.0	50.0		
317.1407	317.1414	-0.7	-2.2	9.5	n/a	C16 H18 N4 O2 F
	317.1400	0.7	2.2	4.5	n/a	C15 H22 O6 F
	317.1402	0.5	1.6	13.5	n/a	C19 H17 N4 O

6-Isopropoxy-9-isopropyl-9*H*-purine (**MJ-16**). ^{1}H NMR (300 MHz, $CDCl_3$) δ 8.51 (s, 1H), 7.95 (s, 1H), 5.67 (p, *J* = 6.4 Hz, 1H), 4.88 (p, *J* = 6.8 Hz, 1H), 1.62 (d, *J* = 6.8 Hz, 9H), 1.47 (d, *J* = 6.1 Hz, 10H). ^{13}C NMR (75 MHz, $CDCl_3$) δ 160.76, 151.95, 150.17, 139.52, 122.18, 70.34, 47.47, 22.81, 22.16. HRMS (ES + ve), $C_{11}H_{17}N_4O$ $(M + H)^+$: Calculated 221.1402. Obtained 221.1402.

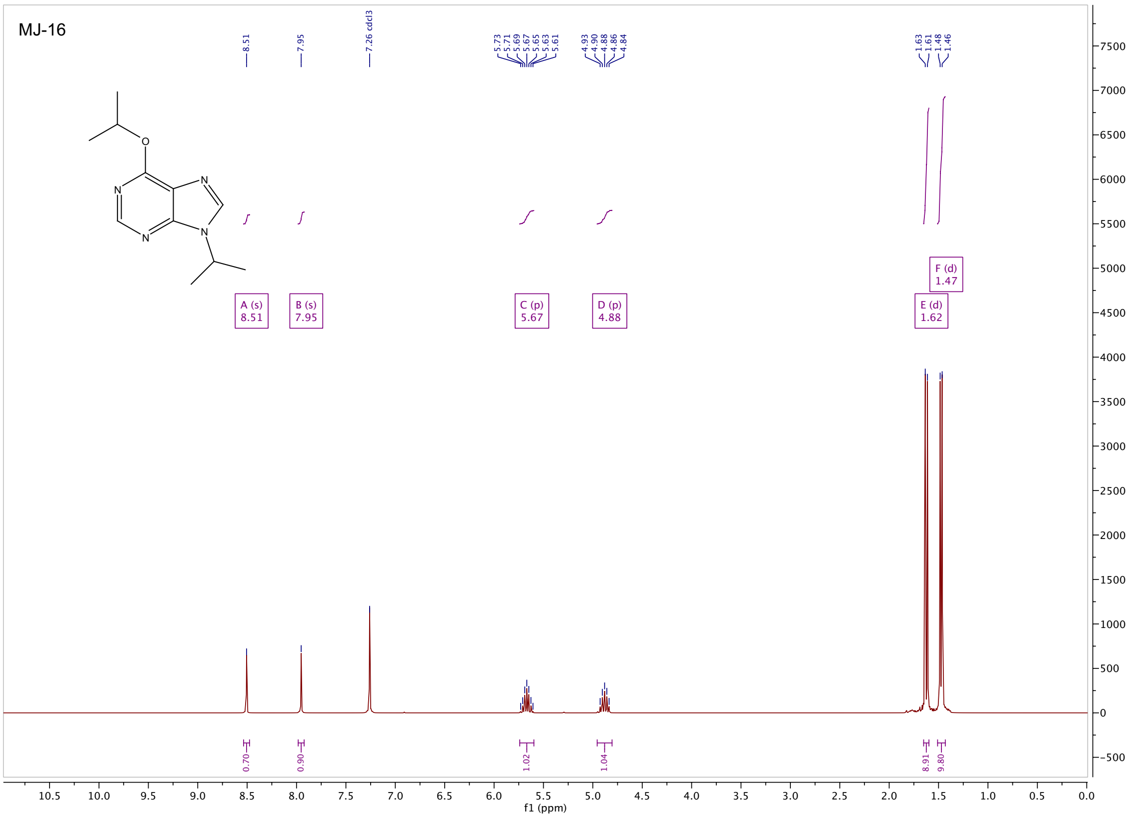

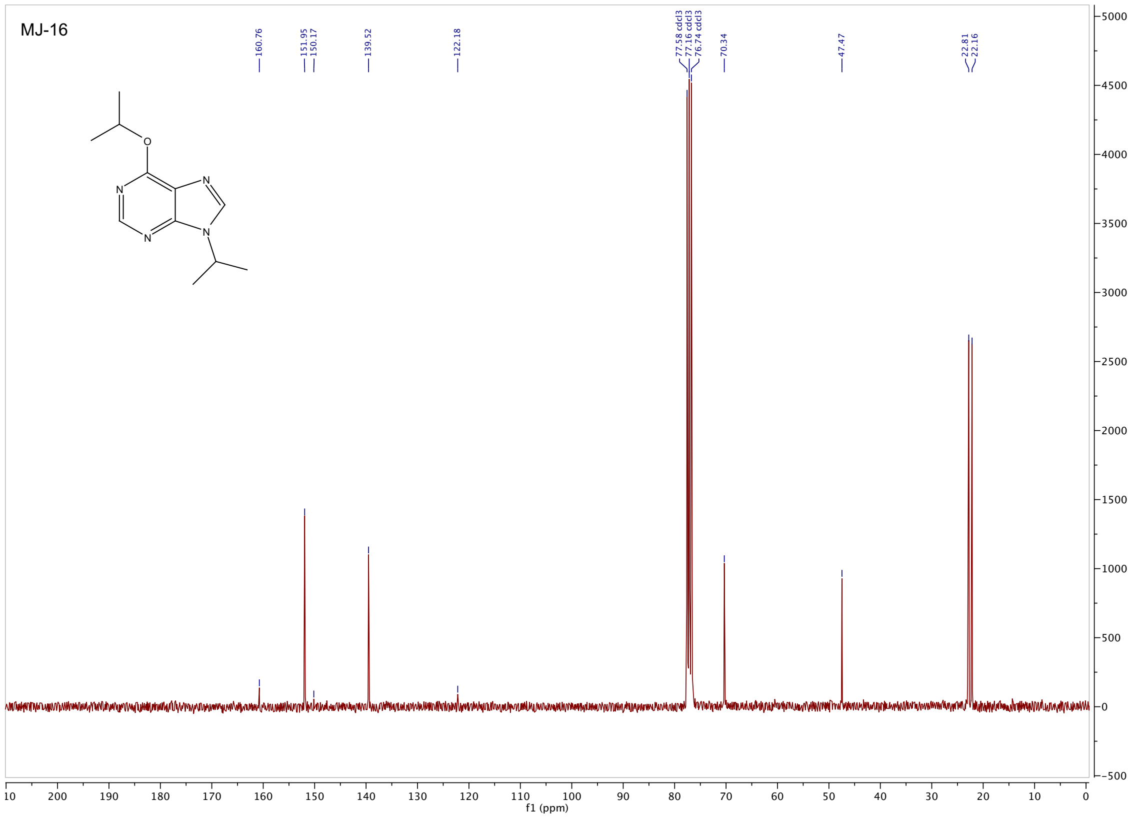

5j. – 6-Isopropoxy-9-isopropyl-9H-purine (MS)

Elemental Composition Report **Page 1**

Single Mass Analysis
Tolerance = 5.0 PPM / DBE: min = -1.5, max = 50.0
Element prediction: Off
Number of isotope peaks used for i-FIT = 3

Monoisotopic Mass, Even Electron Ions
422 formula(e) evaluated with 1 results within limits (up to 50 closest results for each mass)
Elements Used:
C: 0-15 H: 0-1000 N: 0-10 O: 0-10 Na: 0-1

12/7270

ASIMJ-16 69 (1.534) 1: TOF MS ES+ 3.64e+002

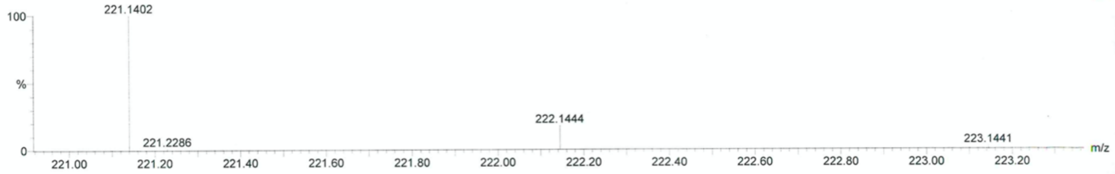

Minimum:				-1.5		
Maximum:		20.0	5.0	50.0		
Mass	Calc. Mass	mDa	PPM	DBE	i-FIT	Formula
221.1402	221.1402	0.0	0.0	5.5	2.0	C11 H17 N4 O

6-Isopropoxy-9-isopropyl-8-methyl-9*H*-purine (**MJ-17**). ^{1}H NMR (300 MHz, $CDCl_3$) δ 8.50 (s, 1H), 5.65 (ddt, J = 12.5, 11.0, 6.2 Hz, 1H), 4.88 (p, J = 6.8 Hz, 1H), 2.64 (s, 3H), 1.62 (d, J = 6.8 Hz, 6H), 1.47 (d, J = 6.2 Hz, 6H). HRMS (ES + ve), $C_{12}H_{19}N_4O$ $(M + H)^+$: Calculated 235.1559. Obtained 235.1553.

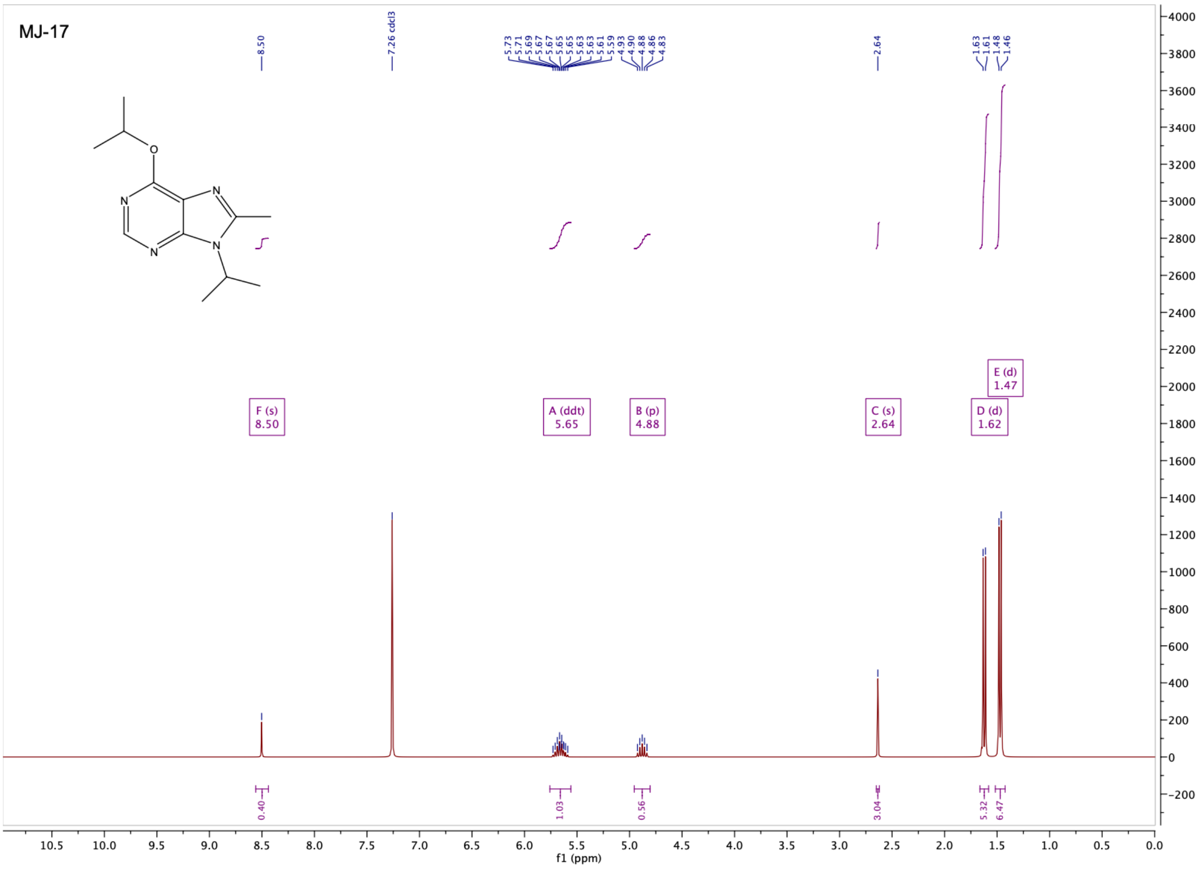

Elemental Composition Report Page 1

Single Mass Analysis
Tolerance = 5.0 PPM / DBE: min = -1.5, max = 50.0
Element prediction: Off
Number of isotope peaks used for i-FIT = 3

Monoisotopic Mass, Even Electron Ions
463 formula(e) evaluated with 2 results within limits (up to 50 closest results for each mass)
Elements Used:
C: 0-15 H: 0-1000 N: 0-10 O: 0-10 Na: 0-1

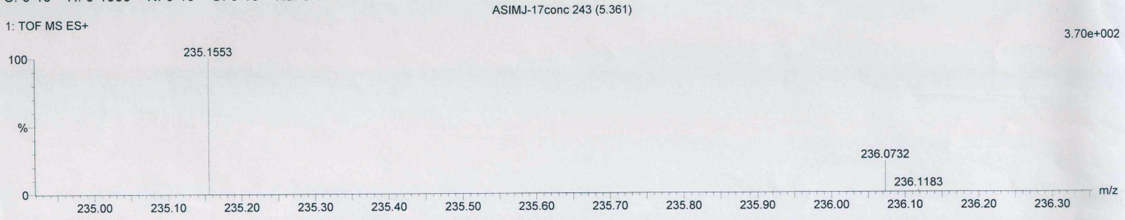

Minimum:				-1.5		
Maximum:		20.0	5.0	50.0		
Mass	Calc. Mass	mDa	PPM	DBE	i-FIT	Formula
235.1553	235.1559	-0.6	-2.6	5.5	n/a	C12 H19 N4 O
	235.1545	0.8	3.4	0.5	n/a	C11 H23 O5

9-*tert*-Butyl-6-ethoxy-9*H*-purine (**MJ-24**). ^{1}H NMR (300 MHz, $CDCl_3$) δ 8.50 (s, 0H), 7.98 (s, 1H), 4.65 (q, J = 7.0 Hz, 2H), 1.81 (s, 9H), 1.51 (t, J = 7.1 Hz, 3H). ^{13}C NMR (75 MHz, $CDCl_3$) δ 161.12, 151.10, 149.92, 139.78, 136.06, 123.07, 63.01, 57.70, 29.21, 14.70. HRMS (ES + ve), $C_{11}H_{17}N_4O$ (M + H)$^+$: Calculated 321.1402. Obtained 321.1400.

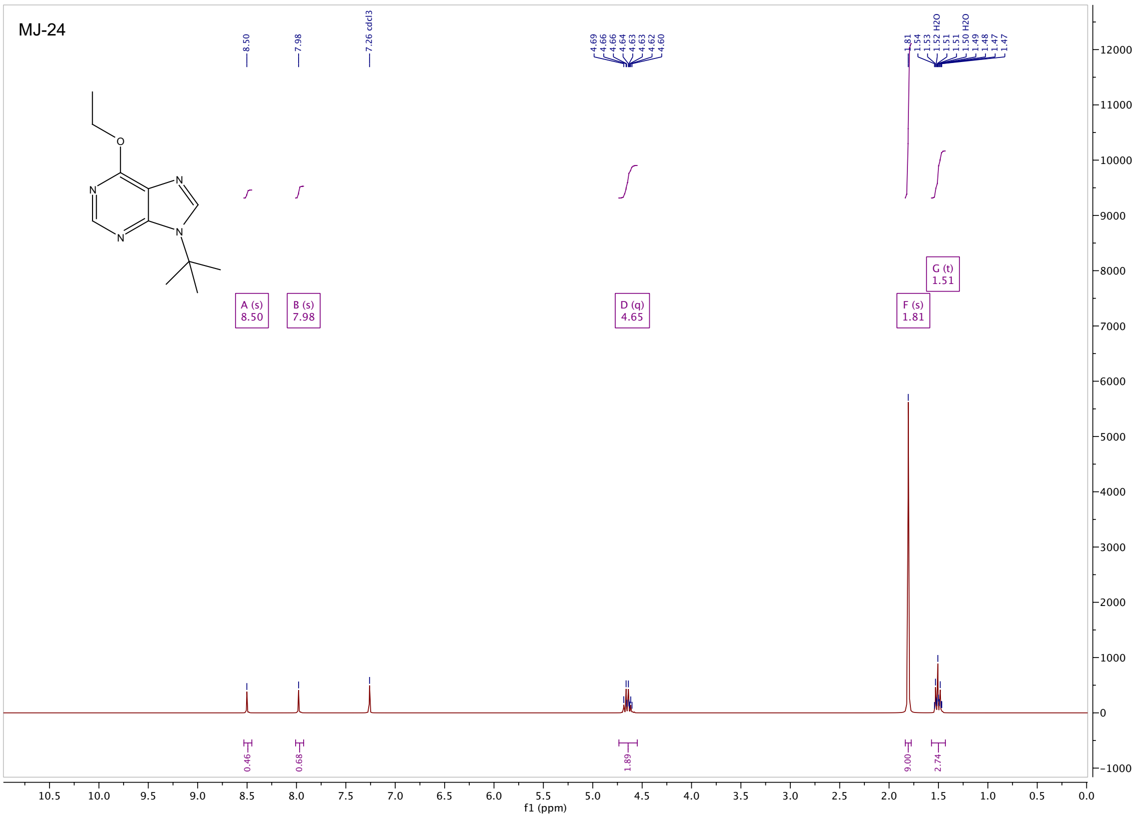

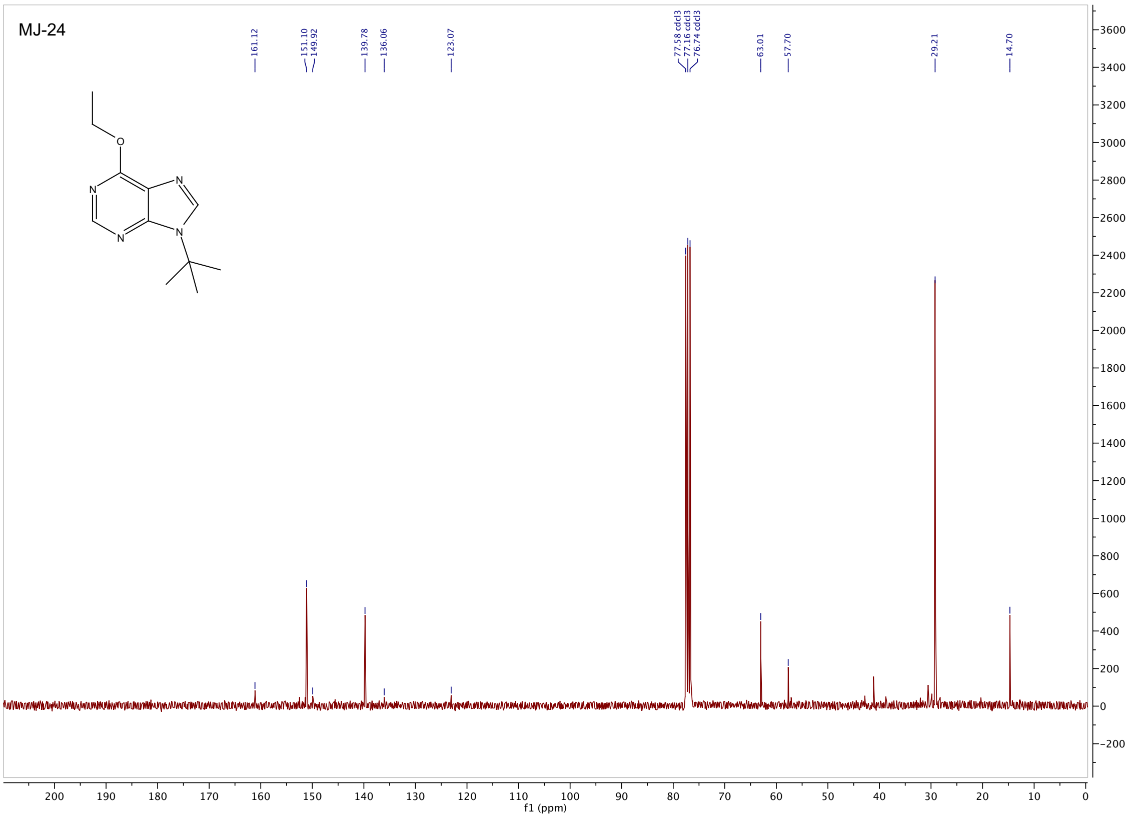

5n. - 9-tert-butyl-6-ethoxy-9H-purine (MS)

Elemental Composition Report **Page 1**

Single Mass Analysis
Tolerance = 5.0 PPM / DBE: min = -1.5, max = 50.0
Element prediction: Off
Number of isotope peaks used for i-FIT = 3

Monoisotopic Mass, Even Electron Ions
362 formula(e) evaluated with 2 results within limits (up to 50 best isotopic matches for each mass)
Elements Used:
C: 0-56 H: 0-1000 N: 0-4 O: 0-10 Na: 0-1 Br: 0-1

ASIMJ-24 8 (0.175)
1: TOF MS ES+

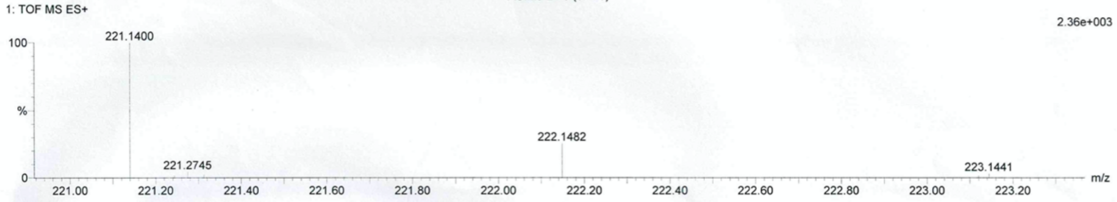

Mass	Calc. Mass	mDa	PPM	DBE	i-FIT	Formula
Minimum:				-1.5		
Maximum:		5.0	5.0	50.0		
221.1400	221.1402	-0.2	-0.9	5.5	66.8	C11 H17 N4 O
	221.1389	1.1	5.0	0.5	88.4	C10 H21 O5

6-(Benzyloxy)-9-phenethyl-8-phenyl-9*H*-purine (**6g**).^{1}H-NMR (400 MHz, $CDCl_3$): δ 8.58 (1H, s, CH), 7.60 – 7.53 (2H, m, 2 x CH), 7.52 – 7.41 (3H, m, 3 x CH), 7.39 – 7.24 (5H, m, 5 x CH), 7.21 – 7.13 (3H, m, 3 x CH), 6.96 – 6.87 (2H, m, 2 x CH), 5.71 (2H, s, OCH_2), 4.56 (2H, t, *J* = 7.4 Hz, CH_2), 3.10 (2H, t, *J* = 7.4 Hz, CH_2). ^{13}C-NMR (126 MHz, $CDCl_3$): δ 161.23 (C), 158.80 (C), 154.40 (CH), 139.32 (C), 137.42 (C), 136.96 (C), 131.17 (CH), 129.89 (C), 129.12 (2 x CH), 128.81 (2 x CH), 128.78 (2 x CH), 128.66 (2 x CH), 128.34 (2 x CH), 128.13 (CH), 127.85 (2 x CH), 126.59 (CH), 111.10 (C), 68.25 (CH_2), 42.36 (CH_2), 36.18 (CH_2). HRMS (ES + ve), $C_{26}H_{23}N_4O$ $(M + H)^+$: Calculated 407.1872. Obtained 407.1848.

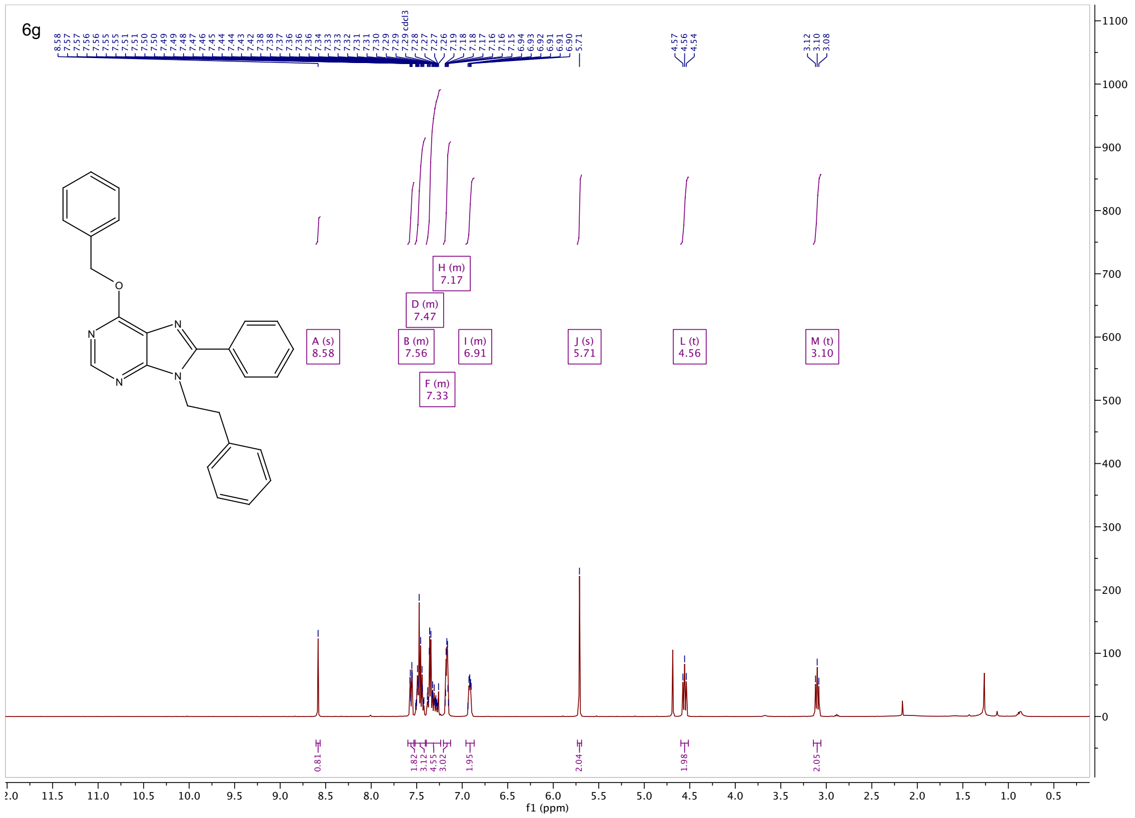

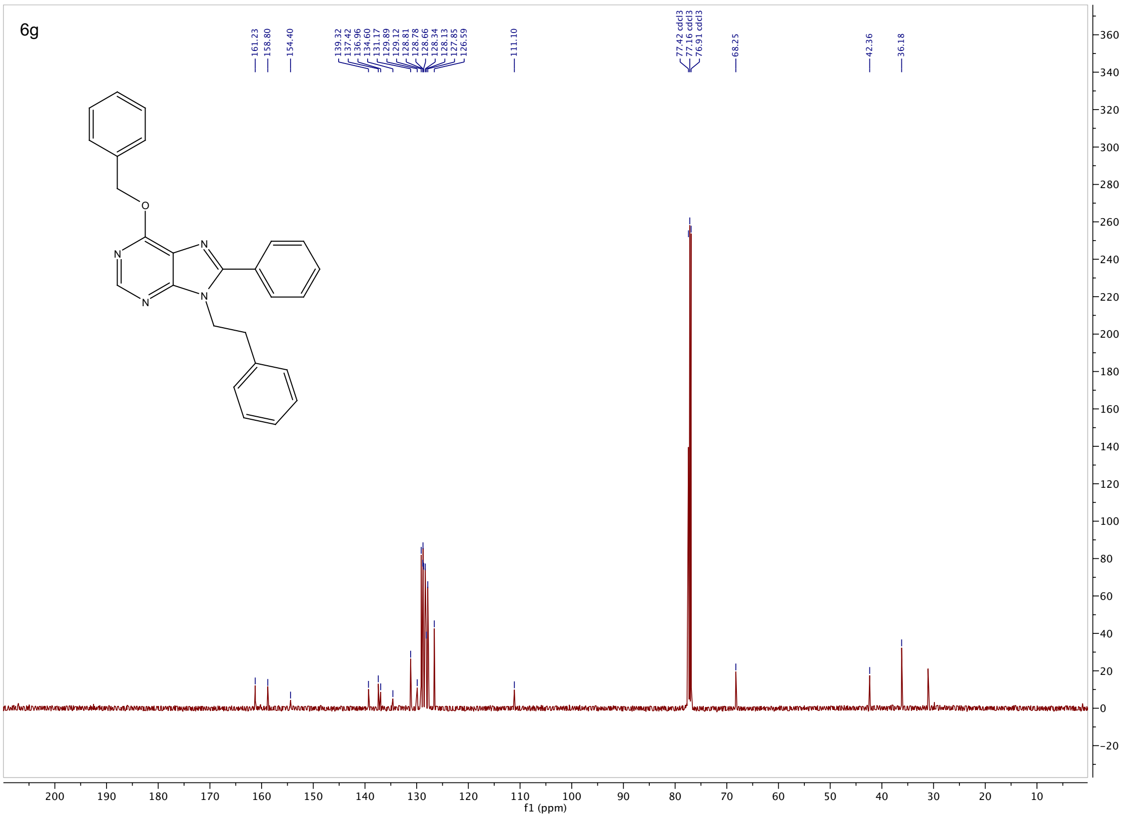

Elemental Composition Report **Page 1**

Single Mass Analysis
Tolerance = 10.0 PPM / DBE: min = -1.5, max = 50.0
Element prediction: Off
Number of isotope peaks used for i-FIT = 3

Monoisotopic Mass, Even Electron Ions
783 formula(e) evaluated with 8 results within limits (up to 50 closest results for each mass)
Elements Used:
C: 0-26 H: 0-1000 N: 0-9 O: 0-9 Na: 0-1
MBQ-I 186 (4.071) AM (Top,5, Ht,5000.0,0.00,1.00)
1: TOF MS ES+

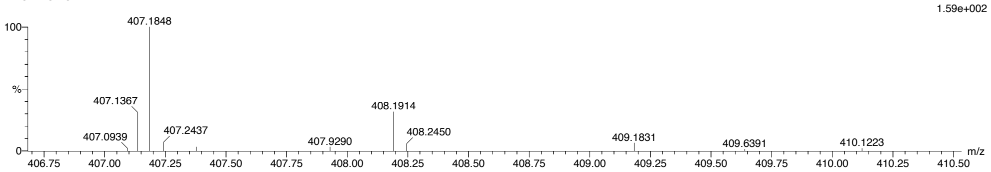

Mass	Calc. Mass	mDa	PPM	DBE	i-FIT	i-FIT (Norm)	Formula
Minimum:				-1.5			
Maximum:		5.0	10.0	50.0			
407.1848	407.1872	-2.4	-5.9	17.5	25.9	1.6	C26 H23 N4 O
	407.1858	-1.0	-2.5	12.5	25.6	1.3	C25 H27 O5
	407.1848	0.0	0.0	14.5	25.8	1.5	C24 H24 N4 O Na
	407.1834	1.4	3.4	9.5	26.3	1.9	C23 H28 O5 Na
	407.1832	1.6	3.9	13.5	26.6	2.3	C21 H23 N6 O3
	407.1818	3.0	7.4	8.5	27.5	3.2	C20 H27 N2 O7
	407.1808	4.0	9.8	10.5	28.5	4.2	C19 H24 N6 O3 Na
	407.1866	-1.8	-4.4	1.5	28.6	4.3	C12 H28 N6 O8 Na

9-*tert*-butyl-6-(benzyloxy)-8-phenyl-9*H*-purine (**6D**).^{1}H NMR (300 MHz, $CDCl_3$) δ 8.55 (s, 1H), 7.58 – 7.51 (m, 2H), 7.44 (q, J = 6.4, 5.5 Hz, 5H), 7.37 – 7.27 (m, 3H), 5.65 (s, 2H), 1.66 (s, 9H). ^{13}C NMR (75 MHz, $CDCl_3$) δ 160.52, 154.43, 153.27, 150.36, 136.48, 134.87, 130.01, 129.62, 128.75, 128.48, 128.17, 127.99, 121.66, 68.33, 60.95, 31.04. HRMS (ES + ve), $C_{22}H_{23}N_4O$ $(M + H)^+$: Calculated 359.1872. Obtained 359.1875.

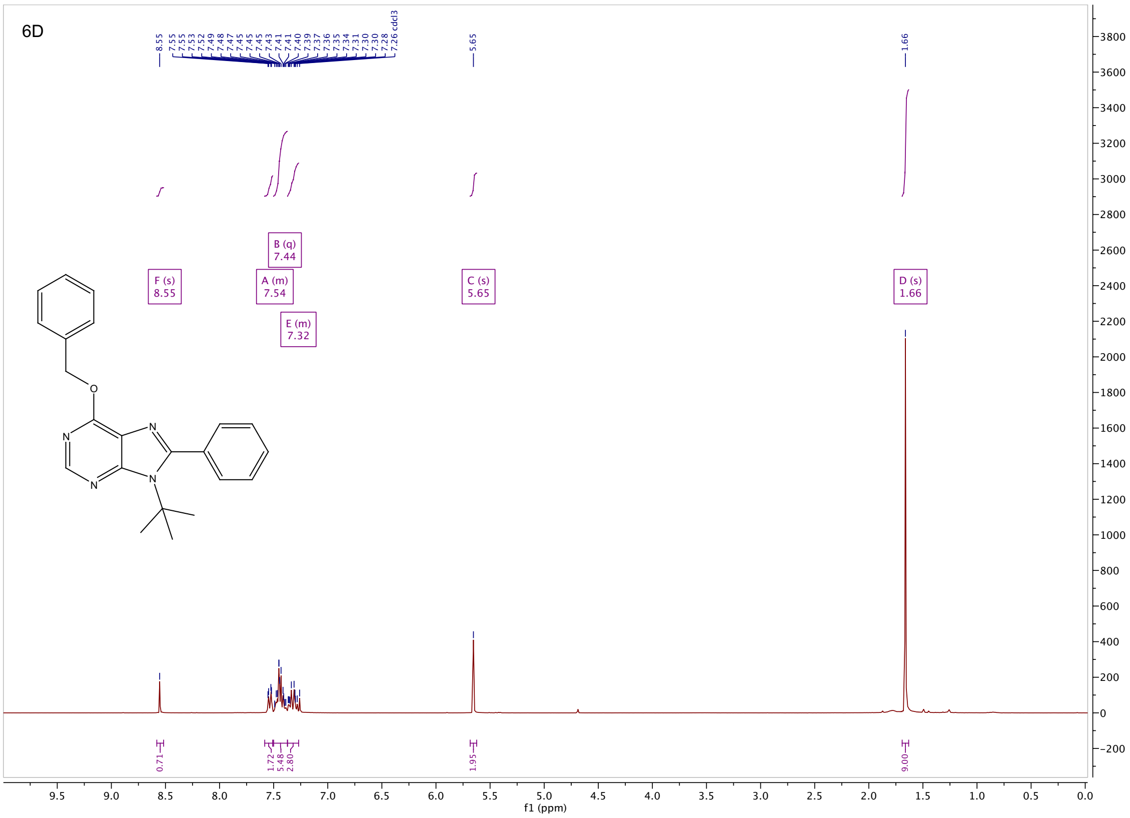

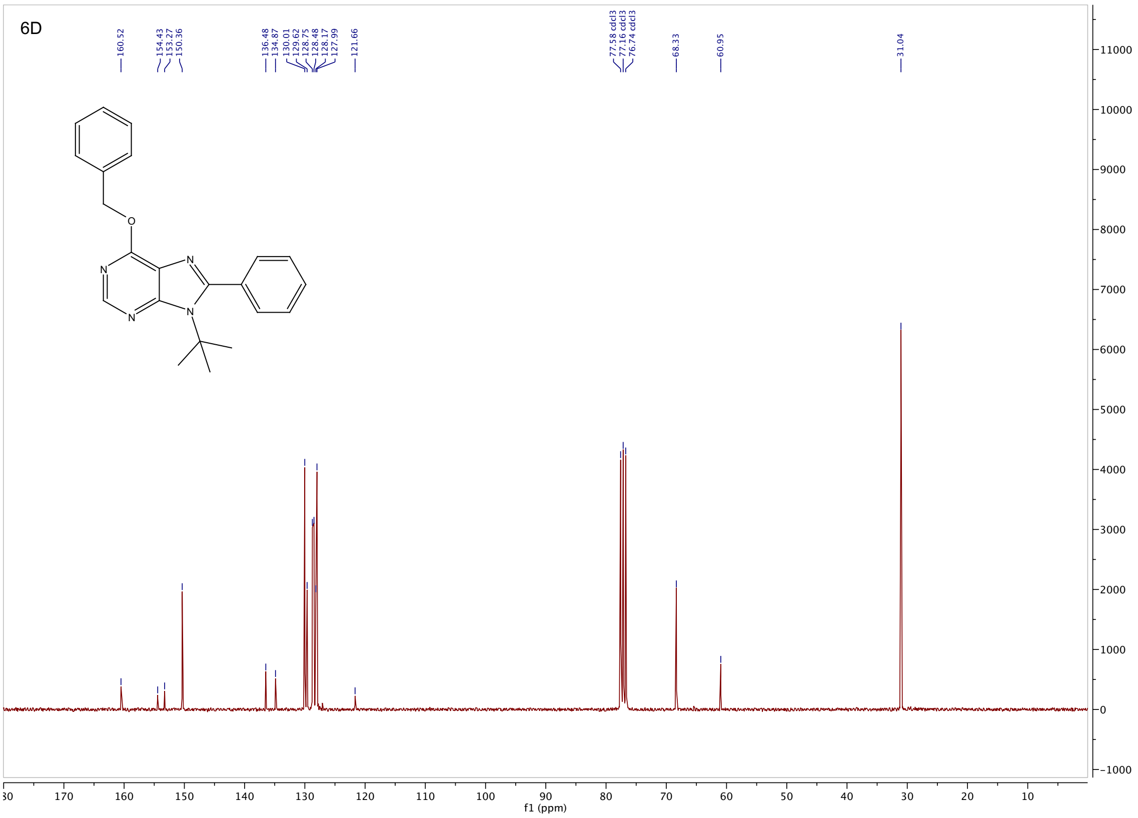

Elemental Composition Report **Page 1**

Single Mass Analysis
Tolerance = 10.0 PPM / DBE: min = -1.5, max = 50.0
Element prediction: Off
Number of isotope peaks used for i-FIT = 3

Monoisotopic Mass, Even Electron Ions
635 formula(e) evaluated with 4 results within limits (up to 50 closest results for each mass)
Elements Used:
C: 0-22 H: 0-1000 N: 0-5 O: 0-20 Na: 0-1
ASIMJ-27 9 (0.195) AM (Cen,5, 40.00, Ht,5000.0,0.00,1.00)
1: TOF MS ES+

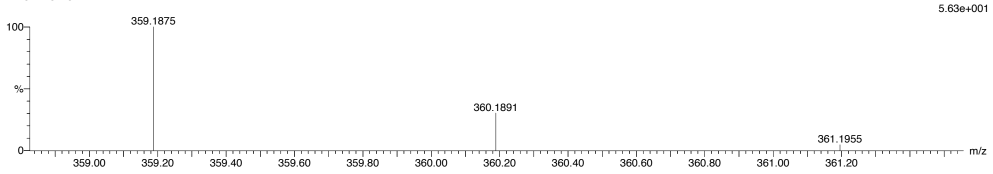

Mass	Calc. Mass	mDa	PPM	DBE	i-FIT	i-FIT (Norm)	Formula
Minimum:				-1.5			
Maximum:		5.0	10.0	50.0			
359.1875	359.1872	0.3	0.8	13.5	5.4	0.9	C22 H23 N4 O
	359.1858	1.7	4.7	8.5	5.7	1.2	C21 H27 O5
	359.1848	2.7	7.5	10.5	6.0	1.5	C20 H24 N4 O Na
	359.1907	-3.2	-8.9	1.5	7.3	2.8	C13 H28 N4 O6 Na
